# A Study on Autonomous Driving Motion Sickness from the Perspective of Multimodal Human Signals

**DOI:** 10.3390/s26051675

**Published:** 2026-03-06

**Authors:** Su Young Kim, Yoon Sang Kim

**Affiliations:** 1 BioComputing Lab, Department of Computer Engineering, Korea University of Technology and Education (KOREATECH), Cheonan 31253, Republic of Korea; sgkim6326@gmail.com; 2 Institute for Bioengineering Application Technology, Korea University of Technology and Education (KOREATECH), Cheonan 31253, Republic of Korea

**Keywords:** autonomous driving, motion sickness, unified sickness, multimodal sensors, signal processing, systematic feature extraction, physiological and behavioral signals

## Abstract

In autonomous driving, motion sickness (MS) arises from physical or visual stimuli, or a combination of both. However, objective quantification of MS level (MSL) remains limited beyond questionnaire-based assessments. Using multimodal human signals (physiological and behavioral) collected in an autonomous driving simulator, this study addresses the association between these signals and MSL, across these MS types, by (i) screening and curating a decade of human-signal MS studies (HS-Set) to establish a data-driven foundation for selecting target sensor domains and features, (ii) constructing a dataset with subjective measures of MSL (fast motion sickness scale and simulator sickness questionnaire (SSQ)), alongside human signals (electroencephalogram (EEG), photoplethysmogram (PPG), electrodermal activity (EDA), skin temperature, and head/eye movement), (iii) conducting a correlation analysis between MSL and the identified features from HS-Set, and (iv) quantifying multivariable contributions at the feature and sensor domains through an explainable boosting machine (EBM). Key correlations include head amplitude/energy (pitch/surge) with SSQ total/oculomotor, eye entropy with nausea/oculomotor (positive), and EDA with nausea (negative). The EBM-based contribution analysis highlights EEG connectivity and head kinematics as dominant contributors; excluding EEG, the interpretability of single-domain models remains limited. Additionally, a combination of Head, PPG, and EDA domains retains over 80% of the full model’s interpretability.

## 1. Introduction

An autonomous vehicle (AV) is a vehicle type in which onboard systems perform driving-related tasks (DRTs), such as steering, accelerating, and braking, thereby transforming drivers into passengers. SAE International has defined six levels of driving automation (level 0–5) in its J3016 standard [1], where higher levels indicate greater automation and reduced driver involvement. For example, a level 2 (L2) vehicle assists with both steering and speed control, but requires constant supervision by the driver, whereas level 4 (L4) and higher vehicles handle all DRTs within their operational design domain. Currently, Tesla provides L2 AVs through its Full Self-Driving software, whereas Waymo operates Waymo One, an L4 ride-hailing service, in Los Angeles, USA. As AV commercialization progresses, drivers are expected to spend more time and engage more frequently in non-driving-related tasks (NDRTs) with increasing levels of automation [2,3,4,5,6].

In their new role as passengers, these tasks will primarily be display-based, such as using handheld displays [6,7,8,9] or watching integrated displays [5,6]. In recent years, these display devices have evolved into see-through head-mounted displays (HMDs) such as Apple Vision Pro and Meta-Orion, a trend that has led to a corresponding increase in research on in-vehicle HMD usage [5,6,10,11].

However, engaging in display-based NDRTs within moving vehicles has the serious side effect of inducing motion sickness (MS) [12,13,14,15]. MS can manifest as classical MS (CMS) from physical stimuli, such as vehicle movement (some studies refer to it as motion-induced MS), visually induced MS (VIMS) from visual stimuli, such as displayed content, or composite MS (Co-MS), resulting from a sensory conflict caused by both stimuli. Although various approaches have attempted to identify the causes of MS and develop mitigation strategies by addressing CMS and VIMS separately or concurrently, the evidence remains fragmentary, and the mechanisms of MS have not been fully elucidated yet.

Quantification of the motion sickness level (MSL) is essential for MS reduction. Conventionally, research has predominantly quantified MSL using subjective questionnaires, including the simulator sickness questionnaire (SSQ) [16], fast motion sickness scale (FMS) [17], and misery scale (MISC) [18]. While these subjective questionnaires have the advantage of being applicable to a wide range of MS types, they have limitations in terms of reliability owing to their dependence on the subjective judgments of the respondents. Some studies on CMS have used the motion sickness dose value (MSDV) (ISO 2631-1) [19], which objectively quantifies ride discomfort that is indicative of MS from vehicle or head acceleration [20,21,22,23]. However, MSDV is limited to representing only CMS, not VIMS or Co-MS.

To overcome these limitations, research has been conducted to quantify MSL using objective human signals, including physiological and behavioral data. However, among these MS studies, those that consider Co-MS arising from display-based NDRTs in moving vehicles are insufficient (see Section 2). Furthermore, machine-learning (ML) and deep-learning (DL) models, which are commonly used to quantify MSL, have a critical limitation: their black-box nature makes them difficult to interpret.

Therefore, in this study, multimodal human signals (physiological and behavioral) collected in an autonomous driving simulator have been used to address the quantitative association between these signals and MSL, a relationship that generalizes across CMS, VIMS, and Co-MS. This has been achieved by (i) systematizing human-signal MS studies from the last decade (2015–2024) to organize handcrafted features by sensor domain, (ii) constructing a dataset by simultaneously collecting subjective MSL measures, four physiological signals, and two behavioral signals, (iii) conducting correlation analysis between MSL and the identified features from the HS-Set, and (iv) quantifying multivariable contributions at the feature and sensor-domain levels using an explainable boosting machine (EBM), to identify interpretable multimodal markers of MS.

Four physiological signals, namely, electroencephalogram (EEG), photoplethysmogram (PPG), electrodermal activity (EDA), and skin temperature (SKT), and two behavioral signals, namely, head and eye movements, were considered for building the dataset. Subjective MSLs were assessed using SSQ and FMS, along with the motion sickness susceptibility questionnaire (MSSQ) [24] to estimate the individual MS characteristics based on prior experience and the film immersive experience questionnaire (Film IEQ) [25] to measure content immersion levels. CMS was induced using a six-degree-of-freedom (6-DOF) simulator (sway, surge, heave, pitch, yaw, and roll), whereas VIMS was induced by presenting high-optical-flow movies through two types of displays: a see-through HMD and a tablet.

The remainder of this paper is organized as follows: Section 2 screens and curates MS studies using human signals. Section 3 describes the experimental apparatus, consisting of an autonomous driving simulator and an acquisition system for multimodal human signals, and defines MS-inducing factors. Section 4 details the experiments based on these factors. Section 5 analyzes the results of the subjective questionnaire data and the objective human-signal data, and examines the contributions of sensor domains and features to MSL. Section 6 discusses the findings of this study, followed by conclusions and future research directions in Section 7.

## 2. Related Work

### 2.1. MS and Human Signals

Research attempting to explain MS using objective human signals has made steady progress. To establish a data-driven foundation for objective MS evaluation and to identify valid parameters that inform our experimental design, recent studies from the last decade were screened and curated (search period: 1 January 2015, to 31 December 2024). The academic databases used were IEEE Xplore, Web of Science, and ACM Digital Library, with the search keywords “(Motion Sickness OR Cybersickness OR VIMS OR VR sickness) AND (Evaluation OR Assessment OR Quantification) AND (Physiological OR Physiology OR Biometric OR Biosignal OR Objective),” where VR refers to virtual reality. The keyword behavioral was intentionally excluded to minimize search noise from studies with low direct relevance, such as cognitive-behavioral studies. Instead, the methodological core keyword objective was included to comprehensively capture the objective measurement studies, such as MS research based on behavioral signals. The specific search queries used for each academic database are listed in Table S1 in the Supplementary Materials.

The literature search was limited to publications from journals and conferences. Studies that did not attempt to explain MS using objective human signals were excluded. Studies that explained MS based solely on stimulus characteristics (e.g., videos and vehicle acceleration) were also excluded. When a collected publication was a review paper, it was excluded unless an objective MS quantification methodology was suggested. Of the 968 records retrieved through the search, 43 duplicates were removed, and 843 publications were excluded, resulting in 82 studies (last search date: 27 April 2025). Hereafter, the human-signal MS study set (HS-Set) refers to the collection of 82 studies curated in this section.

**Figure 1:**
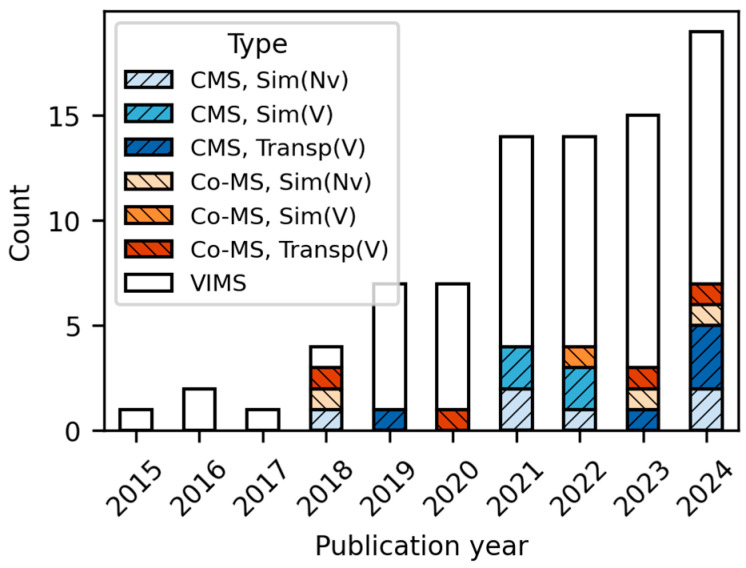
Yearly trends in the HS-Set (Transp = actual transport, Sim = laboratory or simulator settings, V = vehicle, and Nv = non-vehicle).

In the HS-Set studies, CMS cases were classified as simulator-based when physical motion was stimulated, but not in actual transportation. Co-MS cases were limited to studies with separate visual stimuli that were independent of physical motion (e.g., reading a book or watching a display in a moving vehicle). The yearly trends of the classified studies, as shown in Figure 1, were predominantly centered on VIMS, with studies considering CMS and Co-MS gradually emerging since 2018. This trend most likely reflects the rise in data-driven MS quantification approaches, facilitated by advancements in ML/DL technologies in the 2010s and the increasing interest in NDRTs following AV commercialization in the 2020s.

The HS-Set included eight Co-MS studies, four in actual vehicles [11,26,27,28], and four in laboratory or simulator settings [29,30,31,32]. In each study, Co-MS was analyzed in conjunction with various human signals or functional test outcomes. Some studies focused on functional test outcomes to diagnose or predict MS susceptibility. For example, functional head impulse tests (fHIT) and dynamic visual acuity were reported to reflect MS susceptibility and abnormalities in visual-vestibular integration outside transportation simulators [30,31]. In contrast, the vestibulo-ocular reflex (VOR) measured in an actual vehicle showed no significant differences between the susceptible and non-susceptible groups, indicating its limitation as a standalone predictor [26]. Crucially, these approaches focused on diagnostic testing rather than proposing quantitative metrics derived directly from continuous human signals.

Other studies collected continuous physiological signals alongside subjective MS questionnaires to evaluate specific experimental conditions. These studies measured signals such as PPG, EDA, electrocardiogram (ECG), and electrogastrogram (EGG) alongside the SSQ under various conditions in actual vehicles (e.g., different types of VR content, scent conditions, or synchronization levels) [11,27,28]; nevertheless, a common limitation of these studies is that the subjective scores and physiological signals were only compared across conditions without performing a direct joint analysis between them. Even when associations were examined, as in the study by Tamura et al. [32], who analyzed EDA and disorientation criteria in a flight simulator, quantitative metrics were not proposed.

While some prior research has explored Co-MS, it remains relatively insufficient compared to CMS and VIMS. In particular, only five studies were conducted in driving environments (including actual vehicles and simulators), and among these, only Kojima et al. [29] conducted a joint analysis of the measured MSL and collected human signals. However, this study did not propose a quantitative metric for MS using human signals. Therefore, an integrated MS quantification approach based on human signals that can be applied to Co-MS is necessary.

### 2.2. Sensor Domains and Research Trends in the HS-Set

The human signals utilized in the HS-Set can be classified into several sensor domains based on the data acquisition methods; in this paper, both small-scale measurement sensors and larger equipment are collectively referred to as “sensors.” For example, if a heart rate (HR) feature is collected using a PPG sensor, it can be considered as a part of the PPG domain. Similarly, features such as the breath rate (BR) can be either indirectly calculated from the PPG domain or collected using a dedicated respiration (RSP) sensor. The number of studies classified according to these sensor domains is listed in Table 1.

**Figure 2:**
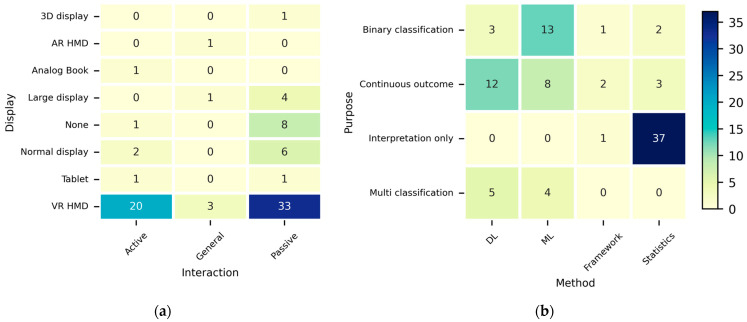
Results of the four-axis categorization (not mutually exclusive, except for method axis): (**a**) Display × Interaction; (**b**) Purpose × Method. The color scale applies to both subgraphs. (AR = augmented reality; other abbreviations are defined in the main text; other abbreviations are defined in the main text).

Summaries of the primary characteristics and utilized human-signal features for each sensor domain are extensively described in Section S1 of the Supplementary Materials (Tables S2–S8). Beyond identifying the types of sensors utilized, understanding the broader experimental contexts in which these signals were collected is crucial for establishing an experimental design. To examine these contextual trends in the HS-Set, the studies were categorized according to four key aspects: ① interaction type, ② display used, ③ research purpose, and ④ approach method. Figure 2 shows the categorization results.

As can be seen from Figure 2a, most studies used VR HMDs, indicating that MS research using human signals over the last decade has predominantly focused on VIMS in VR environments. Normal and large displays were frequently used in eight and five studies, respectively, whereas other display types were used in only a few cases. Importantly, despite the trend toward see-through HMDs, only one study used an AR HMD [80]. This underscores the strong need for Co-MS research that combines see-through HMDs with NDRTs in the context of autonomous driving.

Interaction types were classified into two categories based on whether users could actively control the experimental content or only passively experience it (general was used for studies applicable to both categories). The most common interaction was passive, which could stem from efforts to minimize behavioral confounds to clearly analyze the stimulus–response relationships. Active interaction was also frequently employed to induce MS-related factors, such as cognitive load, by requiring users to perform specific tasks.

Figure 2b shows the approach methods according to the research purpose. Studies that involved calculating continuous values through various models (theoretical, statistical, and neural networks) were classified as continuous outcomes, those that involved distinguishing MS-related states at two levels were classified as binary classification, and those with three or more levels were classified as multi-classification. Studies that did not fit into these categories, such as those that focused solely on interpretation, were classified as interpretation only.

Unlike the other axes, the approach method axis did not allow for overlapping classifications; the studies were classified based on their core approach method. Studies that focused on statistical models or analyses were classified as statistics, whereas those proposing new methodologies (e.g., metrics, theoretical models, and architectures) were classified as frameworks. Among learning-based studies, those employing neural network models were classified as DL, whereas those with shallow networks were classified as ML.

Most studies in the HS-Set were classified as interpretation only × statistics. This indicates that because MS mechanisms have not yet been clearly elucidated and validation frameworks are insufficient, the predominant approach has been to interpret the phenomenon using statistical methods. ML and DL approaches were also frequently used for prediction and classification despite their lower interpretability. ML was primarily used for binary classification, such as determining the presence of MS, whereas DL was mainly used to predict subjective MSL measures, such as SSQ or FMS, using regression. However, ML- and DL-based prediction problems are limited by their low interpretability owing to their predominantly black-box structures.

**Figure 3:**
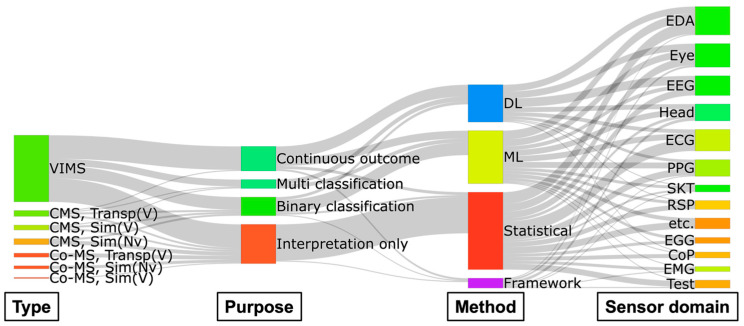
Sankey diagram of the MS type, purpose, method, and sensor domain. (All abbreviations are defined in the main text).

A Sankey diagram illustrating the relationships between the MS type, research purpose, approach method, and sensor domains used is shown in Figure 3.

Figure 3 reveals an imbalance in MS research using human signals. In terms of MS types, research is excessively biased toward VIMS, whereas Co-MS studies are not only sparse but also limited to interpretation only. Among studies not restricted to interpretation only, some attempted to quantify MS through multi-classification, but these were relatively few compared to continuous outcomes and binary classification. The selection of sensor domains was dispersed without consistent patterns for any specific type-purpose-method combination.

These facts imply that (i) the foundation of MSL research on Co-MS is still weak, (ii) sensor selection was likely driven more by availability or convention than by hypotheses, and (iii) there is a need for an integrated platform to analyze the relationships between complex factors (e.g., MS type and display type) and multimodal sensor domains.

To establish such an integrated approach, it is essential to understand how MS is reflected in human signals. The HS-Set suggests that MS is rarely captured using a single metric. Instead, it corresponds to complex variations across multiple domains, including autonomic nervous system responses (e.g., cardiovascular and electrodermal changes), central nervous system activities (e.g., EEG spectral patterns), and behavioral or oculomotor indicators (e.g., head and eye movements). These cross-domain trends provide an essential rationale for adopting a multimodal measurement strategy. Accordingly, based on the primary domains summarized in this section (Table 1 and Section S1), we configured the targeted sensor setup (Section 3) and established a feature extraction framework (Section 4) to enable joint quantitative analyses with subjective MSL measures across CMS, VIMS, and Co-MS.

## 3. Materials

### 3.1. Autonomous Driving Simulator

Collecting data through actual vehicle driving might be the most intuitive approach for quantitatively studying MS in AV environments. However, owing to driving-safety concerns and variable control issues across repeated experiments, it is difficult to provide identical experimental scenarios repeatedly for dozens of participants. Furthermore, because current AVs operate at L4 only in limited regions, there are constraints on the intentional and repeated implementation of various scenarios (e.g., acceleration/deceleration, turning, and slope).

To overcome these limitations, a 6-DOF motion platform was employed to simulate an autonomous driving environment instead of an actual vehicle. The motion platform used was the XTA Pro G6 model from GAMA System in Korea, with maximum motion ranges of sway = ±115 mm, heave = ±120 mm, surge = ±75 mm, pitch = ±33°, yaw = ±20°, and roll = ±28°. The motion platform was installed in a laboratory equipped with window blinds and an air conditioner to minimize environmental factors such as lighting and temperature. It was paired with a large 65-inch display (Samsung Signage Flip) to enhance the visual immersion of passengers.

**Figure 4:**
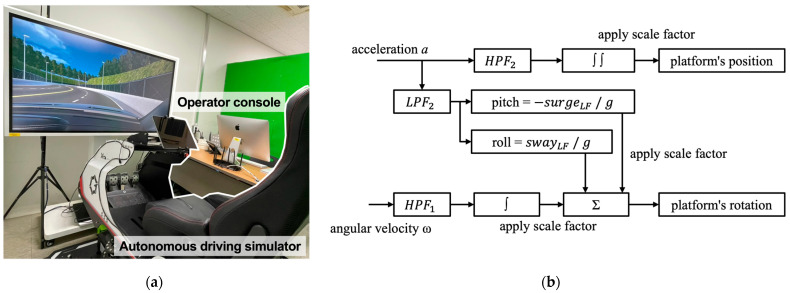
Implemented autonomous driving simulator: (**a**) Experimental setup; (**b**) Motion cueing algorithm based on the classic washout filter applied to the simulator.

Additionally, green partitions were installed within the experimental environment to minimize external visual stimuli, and an operation console was positioned next to the platform to enable real-time control of the experiment and monitor the sensors. Figure 4a shows the setup of the autonomous driving simulator used in the laboratory.

The motion cueing algorithm (MCA) applied to the autonomous driving simulator was the classic washout filter method, as shown in Figure 4b, which is widely used owing to its effective real-time performance [107,108]. Detailed parameter settings of the MCA, including the cutoff frequencies and scale factors for each filter, are provided in Section S2.1 of the Supplementary Materials (Table S9).

The virtual driving content was developed using the Unity engine and EasyRoads3D package, and comprised four driving scenarios: acceleration/deceleration, constant velocity, constant velocity turning, and constant velocity on slopes. The two constant-velocity conditions (turning and slope) refer to a constant linear velocity, not a constant angular velocity, which is similar to the cruise control in actual vehicles. To synchronize with the MCA, each scenario used predefined motion profiles based on acceleration and angular velocity. Detailed procedures for generating these motion profiles and virtual trajectories (e.g., road generation and coordinate transformation) are described in Section S2.2 (Figures S1 and S2).

The virtual driving content excluded surrounding vehicles and traffic signals. The surrounding environment consisted of repeated patterns, namely lamps at regular 25-m intervals and two types of trees at 10-m intervals (with small random offsets to prevent perceptual adaptation to regular patterns), to induce vection related to MS [109]. Based on the 2023 statistics from the Ministry of Land, Infrastructure, and Transport (MOLIT), the road was designed as a four-lane road (two lanes in each direction, with a lane width of 3.5 m), which is the most common configuration for general roads and highways in Korea. In Korea, the first lane is typically the passing lane. Therefore, the second lane was used as the driving lane for the experiment, considering that AVs are expected to travel at a constant speed.

### 3.2. Multimodal Human-Signal Acquisition System

Although the sensor domains utilized in the HS-Set were diverse, some sensors (e.g., center of pressure (CoP), Gait) were unsuitable for driving environments. Additionally, ECG and PPG provided largely overlapping feature information, and a few RSP features could be derived from these sensors. Kenward et al. [110] and Wickham [111] reported that nausea, a major symptom of MS, is a higher-level cognitive activity than vomiting. Kim et al. [47] found no significant association between electromyogram (EMG), which belongs to the somatic nervous system rather than the autonomic nervous system, and MS. Considering these factors, the EGG, EMG, CoP, and Gait domains were excluded. PPG was selected for cardiovascular sensing instead of ECG because of the ease of wearability of the PPG sensor, and no dedicated RSP sensor was used because RSP data could be indirectly estimated from the ECG or PPG signals.

The brain activity can usually be measured using various sensor domains such as EEG, functional near-infrared spectroscopy (fNIRS), and functional magnetic resonance imaging (fMRI). However, EEG data were used in 20 studies in the HS-Set, whereas fNIRS and fMRI data were used in only three or fewer studies each. In particular, because fMRI relies on large and fixed equipment, it cannot be applied in driving environments. Moreover, wearing both fNIRS and EEG sensors can cause not only physical interference but also reduced immersion, leading to cumulative fatigue. Thus, only the EEG was selected for measuring the brain activity.

Impedance cardiography (ICG) requires direct electrode attachment to the chest, which can be burdensome for the participants and might cause ethical or psychological discomfort. As these constraints could reduce data reliability, ICG was also excluded. Blood pressure (BP) applies physical pressure to the body, which could divert the attention of the participants from this measurement. This increases the likelihood of bodily responses being distorted by the external factor of pressure rather than by the MS-inducing effects. Therefore, BP was also excluded. The endocrine and assessment test domains were not considered because they were difficult to use as real-time signals. Following this selection process, the human-signal sensors selected for this acquisition system are listed in Table 2.

**Figure 5:**
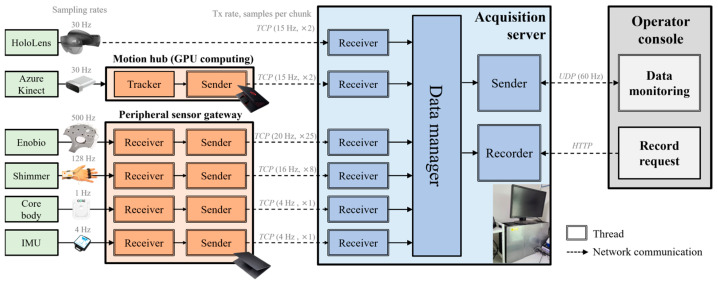
Multimodal human-signal acquisition system. Motion hub = Samsung Odyssey (GPU: GTX 1060), peripheral sensor gateway = Lenovo ThinkPad Gen1, acquisition server = HP Z820 (OS: Windows 10). The core body temperature sensor was sampled at 1 Hz but transmitted at 4 Hz to avoid dropouts from 1 Hz server logging.

The selected sensors had different sampling rates, making time synchronization essential for simultaneous collection. To address this issue, a multimodal human-signal acquisition system was implemented to collect the sensor data in parallel and align them along a common time axis. As shown in Figure 5, the acquisition system was designed with multithreading for parallel processing and included two subsystems: a motion hub that distributed GPU-accelerated body-tracking computations, and a peripheral sensor gateway that collected and transmitted data from peripheral sensors lacking communication modules.

To ensure stable parallel recording of multimodal signals with varying sampling rates (up to 500 Hz), the data from each sensor were buffered and time-aligned to a common clock referenced to the EEG stream every second. Further engineering details regarding data synchronization and system I/O optimization are described in Section S3, and the operator console interface is illustrated in Figure S3.

### 3.3. Design of MS-Inducing Scenarios

For both CMS and VIMS, the MS symptoms can intensify with longer stimulus exposure times [112,113,114,115,116,117]. Although MS can be induced with just 10–15 min of stimulus exposure [113,116,118], it can also be reduced by adaptation to prolonged repeated stimuli [115,119]. Accordingly, the scenarios were designed with a 20 min stimulus exposure time to induce MS while providing nonrepetitive stimuli.

CMS can be induced by rotation, acceleration/deceleration, and vertical movements [120,121,122,123,124]. According to the sensory conflict theory, the vestibular system acts as a biological accelerometer that detects angular acceleration (semicircular canals) and linear acceleration (otoliths). Therefore, velocity changes amplify sensory conflict and induce MS. CMS can also be caused by vehicle motion that produces optic flow expansion or contraction in the field of view of a passenger [125,126]. Additionally, although not a physical motion, MS in virtual driving environments has been reported to be maximized at a perceived speed of 10 m/s [127]. Considering these CMS-inducing factors, the basic driving speed was set to 40 km/h (rounded up from 36 km/h ≈10 m/s for convenience), and CMS-inducing driving scenarios were designed using the implemented simulator (Table 3).

**Figure 6:**
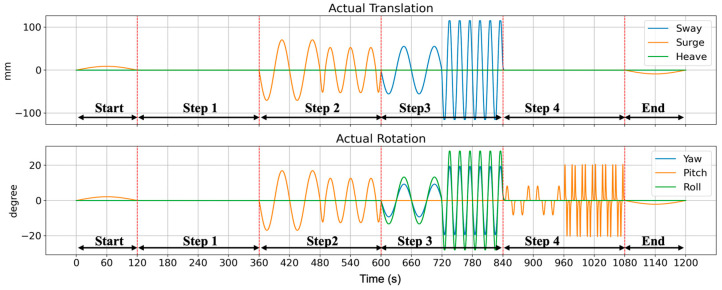
Actual motion of the autonomous driving simulator under the designed CMS-inducing scenarios. The vertical red dashed lines denote the boundaries between different scenario steps.

The designed scenarios consisted of four steps (excluding the start and end steps) to prevent participants from adapting to repetitive stimuli. The intensities of the stimuli were varied within each step to further prevent this adaptation. Figure 6 shows the actual motion of the simulator under the designed driving scenarios.

Modern VIMS can be categorized into two types: MS induced by content on traditional flat displays (e.g., tablets and laptops) and MS induced by content on HMDs. For these categories, an Apple iPad Pro (12.9-in 6th generation) and a Microsoft HoloLens 2 were used in this study. The reason for adopting a see-through HMD rather than a VR HMD was to reflect the trend of next-generation displays evolving toward see-through HMDs and to address the significant research gap in AR HMD studies using human signals. Furthermore, the HoloLens 2 provides native head and eye sensor data, allowing it to be used with the implemented acquisition system without additional hardware changes. Hereafter, the VIMS conditions corresponding to flat-panel displays and see-through HMDs are referred to as F-VIMS and S-VIMS, respectively.

The causes of VIMS have primarily been studied from the perspective of content, with reports indicating that it occurs when the content has a high optical flow (OF) or induces fast vection [127,128,129,130]. Additionally, although immersion increases when fidelity, the degree to which reality is well reproduced, is higher, it can also induce VIMS more strongly than lower fidelity [128,131,132]. Accordingly, to provide high OF and fast vection, films from the live-action category that employed subjective camera technique, which is characterized by high fidelity owing to the use of real-world content, were selected in this study. Since this camera technique is predominantly used in the found footage genre, the initial pool of candidate films was selected from the Internet Movie Database (IMDb) based on the criteria of being a movie in this genre, having more than 100,000 votes, and being released after 2000.

From the initial pool of candidates, films that did not use the subjective camera technique were excluded, and films rated as severe for sex and nudity according to IMDb’s parental guidance were also excluded to adhere to research ethics. To ensure that the experimental results were not dependent on a specific video, two films with high OF were selected from the filtered candidates. For this, the DVD version of each film was converted to grayscale, and the mean magnitude of the OF vectors over 20 min was calculated using the OpenCV Farnebäck method (parameters: pyramid scale = 0.5, number of levels = 3, averaging window size = 15 px, iterations per level = 3, neighborhood = 5 px, Gaussian smoothing σ = 1.2, no additional flags). The results showed that End of Watch (2012, OF = 2.924) and Cloverfield (2008, OF = 2.597) had high mean OF values and were selected as VIMS-inducing videos. Although Chronicle (2012, OF = 2.759) had a higher overall OF than Cloverfield, it was not used because its mean OF in the 10–20 min interval was lower (${OF}_{10-20\;min}^{cloverfield}$ = 2.590 and ${OF}_{10-20\;min}^{chronicle}$ = 1.884). The first 20 min of the color versions of these two films were used as VIMS-inducing stimuli in the experiment. Copyright compliance for nonprofit experimental use was confirmed through consultation with the Korea Copyright Commission and legal counsel.

In the Co-MS scenarios, the CMS-inducing driving motions and VIMS-inducing videos were presented simultaneously. By superimposing the visual stimuli of the VIMS scenarios onto the physical stimuli defined in the CMS scenarios, the participants experienced sensory conflict from both sources. This condition was intended to replicate the combination of vehicle motion and exposure to visual content that can occur in actual driving environments.

## 4. Experimental Methods

All research procedures described in this section were conducted in accordance with the guidelines of the Declaration of Helsinki and approved by the Institutional Review Board of KOREATECH in advance (approval on 23 April 2024).

### 4.1. Participants

A total of 100 participants (70 males and 30 females) were recruited through the intranet of the Korea University of Technology and Education, and a small amount of compensation was provided to encourage active engagement. The eligibility of participants was restricted to non-computer science students or first-year computer science students, who were expected to have limited experience with IT equipment, such as simulators and HMDs. The experiment was conducted from 16 December 2024, to 23 January 2025, with a maximum of four participants per day.

To minimize the effects of sleep inertia and sleep pressure on the analysis, the wake-up time of each participant was confirmed. Eight participants were excluded because they had been awake for less than 1 h or more than 11 h. Furthermore, two participants who had consumed alcohol on the day before the experiment were also excluded. Consequently, a total of 90 participants (64 males and 26 females; mean age = 22.73, SD = 1.67) were included in the final experiment. The participant cohort consisted of 82 undergraduate and 8 graduate students, all with normal or corrected-to-normal vision. Their baseline susceptibility to motion sickness averaged 2.600 (SD = 0.909) on a four-point scale.

### 4.2. Experimental Procedure

**Figure 7:**
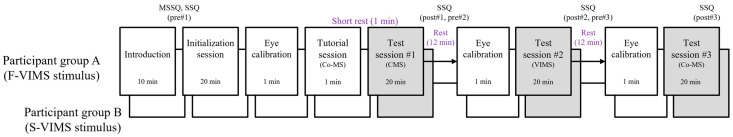
Schematic showing the common experimental procedure for the F-VIMS and S-VIMS groups (approximately 2 h). The two groups follow the identical procedure, differing only in the type of VIMS stimulus. The # symbol denotes the specific ID for each test session (detailed in Section 4.2.2).

The experiment was designed to measure the CMS using the implemented simulator, VIMS using displays, and Co-MS, where both stimuli were applied simultaneously. The experimental procedure was designed as shown in Figure 7 and was conducted by dividing the participants into two groups according to VIMS type (F-VIMS and S-VIMS). Each participant took part in the experiment only once (no crossover participation).

The participants were informed about the experiment, the human signals that would be collected, and the subjective questionnaires for MSL measurements before the experiment. We requested that the participants fill out the subjective questionnaires objectively, mentioning the importance of MSL data in the experiment. Additionally, the characteristics of the found footage genre were briefly introduced to prevent interruptions due to aversion while watching VIMS-inducing content.

**Figure 8:**
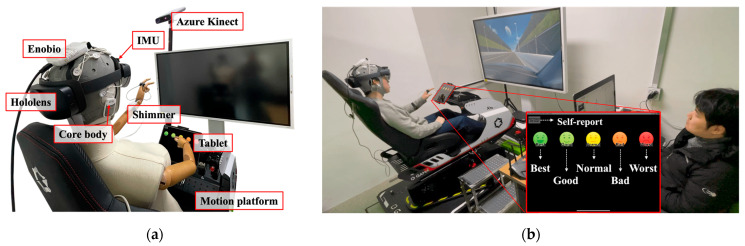
Experimental environment and MSL measurement interface: (**a**) Autonomous driving simulator and multimodal human-signal acquisition system; (**b**) FMS-based measurement interface for MSL input.

In the initialization session, the participants were instructed to take a seat in the simulator and adjust their seats for a comfortable posture. All the sensors of the acquisition system were then attached, and a conductive gel was applied to the EEG sensor electrodes. After confirming that all the sensors were operating correctly through the operator console, the tutorial and test sessions began. Figure 8a shows the implemented simulator and acquisition system, and Figure 8b shows the interface for inputting the FMS for subjective MSL measurement.

#### 4.2.1. Subjective Questionnaires

Subjective MSL was measured using the SSQ and FMS. Additionally, MS susceptibility was assessed using the MSSQ, and content immersion was assessed using the Film IEQ. The SSQ is an MSL assessment tool with 16 items on a four-point scale (0–3), and its results are calculated as subscale scores for three symptom categories: nausea (SSQ_N_), oculomotor (SSQ_O_), and disorientation (SSQ_D_). Weighting factors (SSQ_N_: ×9.54, SSQ_O_: ×7.58, SSQ_D_: ×13.92) are applied to each subscale score, and the total score (SSQ_T_) is calculated as 3.74 times the sum of the unweighted raw subscale scores. The SSQ was administered before eye calibration (pre) and after the test session (post), and the ΔSSQ (post-SSQ score − pre-SSQ score) was used for analysis.

The FMS is an MSL assessment tool that tracks the temporal changes by assessing the current MSL state at regular intervals on a single continuous scale (0–20). In this experiment, the FMS was collected during the test session and modified to use a one-to-five-point scale, as performed in the studies by Wen et al. [133] and Won et al. [119]. The FMS was measured by displaying an evaluation layout on the F-VIMS-inducing device (tablet) at 30-s intervals, as shown in Figure 8b. A notification sound was also played to prevent the S-VIMS participants from failing to recognize it. Furthermore, even if 30 s had not elapsed, the participants were allowed to re-enter their FMS immediately via the self-report button if they experienced a change in their MSL.

The MSSQ estimates the MS susceptibility by assessing the frequency of MS experienced on various transportation and amusement rides during childhood and adulthood. The MSSQ was used to analyze the effect of MS susceptibility on MSL and was administered during the pre-briefing stage.

The Film IEQ is a modified version of the IEQ [134] that assesses the depth of immersion in video games. This modified questionnaire is a tool for evaluating the degree of immersion in a video-watching context, with 24 items rated on a seven-point scale (1–7). The evaluation results are calculated as subscale scores for the four areas through a simple summation of the survey items: captivation (IEQ_cap_), real-world dissociation (IEQ_dis_), comprehension (IEQ_com_), and transportation (IEQ_tra_). In this experiment, the scale was adjusted to a five-point scale to reduce the burden on the participants, and the questionnaire was administered after the test session in which participants viewed the VIMS-inducing videos.

#### 4.2.2. Tutorial and Test Session

The tutorial session was designed to allow participants to preview the stimuli and the FMS measurement method. Participants experienced Co-MS stimuli for 1 min and practiced two types of FMS measurement methods based on 30-s intervals and the self-report button. The CMS stimulus consisted of straight-line driving with 30 s of gradual acceleration followed by 30 s of gradual deceleration (maximum velocity = 40 km/h), and the open-source sample video Big Buck Bunny from Blender was used as the VIMS stimulus. Immediately after the tutorial session, the participants were given a 1 min rest while keeping the sensors on.

The core of the experiment consisted of three test sessions (#1–3). Before each session, the participants wore HoloLens 2 for eye tracking across all conditions (including the F-VIMS condition) and performed a 1 min eye calibration (for Test Session #1, which was performed before the tutorial session). The sessions were conducted in a fixed order, each lasting 20 min: a single CMS stimulus (#1), a single VIMS stimulus (#2), and a Co-MS stimulus (#3).

However, counterbalancing was applied to minimize the adaptation effects of the physical and visual stimuli from #1 and #2, respectively, when conducting #3. A predefined counterbalancing matrix was used across two VIMS types, four driving scenario versions, and two movie orders, yielding 16 combinations (2 × 4 × 2). To maintain balance, participants were sequentially allocated to the next combination in this matrix according to the enrollment order.

The order of the four driving scenario steps (defined in Table 3) was cyclically shifted by one step to create four versions, which were then used to provide different driving scenarios for #1 and #3. For example, participants were assigned to one of four versions based on the starting step of the driving scenario; the first version was driven in the order “Start–1–2–3–4–End” for #1 and “Start–2–3–4–1–End” for #3 (the start and end steps were fixed). In addition, the videos presented in #2 and #3 were counterbalanced by crossing the type and order of the films to control the adaptation effects. For instance, participants were assigned to one of two viewing orders; the first order presented the videos in the sequence “Cloverfield” (#2)– “End of Watch” (#3), while the second order presented them as “End of Watch” (#2)–“Cloverfield” (#3).

After each test session, a 12-min rest period was given to the participants to recover from the MS symptoms [88]. During the rest period, considering the properties of the conductive gel, all sensors except the EEG were temporarily detached and reattached 1 min before the next session. The participants were informed that they could request to stop the experiment at any time if their MS symptoms became severe. After all the experiments were completed, cleaning supplies were provided, and the location of the shower facilities was indicated.

### 4.3. Preprocessing

Although each test session for collecting sensor data lasted 20 min (1200 s), some data were occasionally saved as fragments due to communication and I/O issues during the experiment. For example, if an issue occurred at t = 500 s, the recording was stopped, and the test session was restarted at t = 490 s to resume data collection immediately. These fragmented data were rearranged chronologically and merged, with the overlapping sections trimmed by at least 5 s on each side to reconstruct a continuous 1200 s timeline (e.g., (0–500 s → 0–495 s) + (490–1200 s → 495–1200 s) = 1200 s timeline). This reconstruction process was applied identically, even when three or more data fragments were present. The reconstructed timeline contained unintended noise and outliers at the beginning and end. Therefore, 30 s were trimmed from the start and end of each session, and the remaining 1140 s segment was used as the dataset.

The acquisition system used in the experiment received data in parallel from the sensors with different sampling rates in chunks at approximately 15–20 Hz. All the received data were extracted after every second, synchronized to the EEG (500 Hz) reference time, and saved. During this process, limitations in network and thread scheduling or OS time resolution can cause the actual number of samples in a 1-s interval to deviate from the expected value by approximately ±1 chunk. Consequently, the sensor data in the dataset may contain slight time warping effects.

To correct for this, an expected sample grid was constructed for each sensor based on its official sampling rate multiplied by 1140 s (e.g., for Shimmer3 GSR+, 128 Hz × 1140 s = 145,920 samples). The recorded samples were then mapped to the nearest index on the expected grid. If two or more samples were mapped to the same expected index, the closest sample was assigned, and any empty indices were filled using piecewise cubic Hermite interpolation. Finally, the dataset was corrected by aligning all sensor time series to a common time axis. Although there could be a loss of approximately one chunk (25 samples) of the EEG stream during the storage process of the data manager, this correction procedure reduced the potential for analytical distortion.

**Figure 9:**
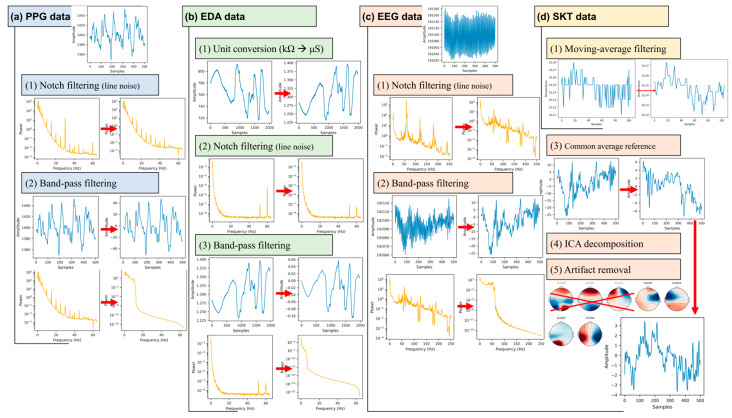
Physiological signal preprocessing pipeline: (**a**) PPG, (**b**) EDA, (**c**) EEG, and (**d**) SKT. The red arrows indicate the sequence of the data processing flow, and the different background colors distinguish the individual sensor domains. The blue and orange line plots represent the signals in the time and frequency domains, respectively.

The PPG, EDA, EEG, and SKT data in the dataset are raw physiological signals that are significantly affected by noise and artifacts. Therefore, they were preprocessed following the procedure summarized in Figure 9. The notch and band-pass filters used for preprocessing were applied using the MNE package 1.9.0 in Python 3.12, and any parameters not explicitly specified were set to the default values of the package.

In the raw PPG data, interference from the Korean power line frequency (60 Hz) and a distinct spectral peak at 30 Hz were observed (Figure 9a, step 1). These two frequency components were suppressed in the frequency domain using a notch filter based on the MNE spectrum-fitting (spectrum_fit) function (all subsequent notch filters were applied using the same method). In addition, the out-of-band noise was suppressed using a Blackman-window-based band-pass filter (all subsequent band-pass filters used the same window), referencing the PPG band used in the previous studies. In the HS-Set, only three studies explicitly reported the PPG band: Dennison et al. [60] used 0.1–10 Hz, Martin et al. [43] used 0.66–3.33 Hz, and Sameri et al. [39] used 0.5–8 Hz. Although these bands partially overlapped, none of the studies used identical bands, and the number of studies was small. Separately, Lapitan et al. [135] reported that a band of 0.1–10 Hz minimizes the pulse waveform distortion in PPG signals, which is identical to the band used by Dennison et al. [60]. Based on this result and the observation of periodic spectral peaks above 10 Hz, as shown in step 2 of Figure 9a, the band-pass range for the PPG preprocessing pipeline was set to 0.1–10 Hz.

While EDA sensors typically measure skin electrical conductance (*G*) in μS, the Shimmer3 GSR+ used in the acquisition system records the electrical resistance (*R*) of the skin in kΩ. As the two physical quantities are related by G = 1/R, each recorded resistance sample, *R*_kΩ_, was converted to the conductance, *G*_μS_ (Figure 9b, step 1). When the EDA data were examined in the frequency domain, unlike the PPG data, no spectral peak was observed at 30 Hz. Therefore, only the 60 Hz component was suppressed using a notch filter (Figure 9b, step 2). In the HS-Set, only the study by Sameri et al. [95] explicitly reported an EDA band of 0.05–4 Hz, and two other studies reported only cutoff frequencies (Lee et al. [38]: upper cutoff 50 Hz and Rahimzadeh et al. [33]: upper cutoff 1 Hz). Therefore, the evidence for setting a band was limited. Separately, Privratsky et al. [136] fixed the upper cutoff frequency of a band-pass filter at 5 Hz and compared various lower cutoff frequency candidates (0.01–0.10 Hz), reporting that 0.02 and 0.03 Hz were effective. Considering that Sameri et al. [39] used an upper cutoff of 4 Hz and Privratsky et al. [136] used 5 Hz, an upper cutoff frequency of 4–5 Hz can be considered a relatively common choice. The band-pass range in the EDA preprocessing pipeline was set to 0.03–5 Hz, which is one of the configurations reported as effective by Privratsky et al. [136] (Figure 9b, step 3). The choice of the lower cutoff frequency was based on the value (0.03 Hz) closer to the 0.05 Hz used by Sameri et al. [39], as opposed to 0.02 Hz.

In the raw EEG data, distinct spectral peaks were observed at the Korean power line frequency of 60 Hz and its harmonics (120, 180, and 240 Hz) (Figure 9c, step 1). These line noise peaks appeared broad owing to the interpolation of lost chunks during the dataset correction process. Therefore, each component was suppressed by setting the notch filter width to 10 Hz (±5 Hz) at 60, 120, 180, and 240 Hz. Most EEG studies in the HS-Set explicitly specified the bands they used, and the reported cutoff frequencies are listed in Table S8.

In the EEG preprocessing pipeline, the lower cutoff frequency for the band-pass filter was set to 1 Hz, which is the most frequently reported value. The upper cutoff frequency was set to 50 Hz. Although 40 and 50 Hz were the most frequently reported values, the former was selected to retain a broader band of information. Step 2 in Figure 9c shows the result of applying the band-pass filter. In addition, a common average reference was applied to suppress the noise common across the entire scalp (Figure 9c, step 3). Subsequently, an independent component analysis (ICA) based on the MNE extended-infomax algorithm was performed (random state = 97), and the MNE-ICALabel model was used to classify and remove non-brain components (e.g., electrooculogram) as artifacts (Figure 9c, steps 4–5).

The raw SKT data had a very low sampling rate of 1 Hz (Nyquist frequency of 0.5 Hz), indicating that the line noise and high-frequency components were not present as resolvable components in the digital domain. Accordingly, no notch or band-pass filters were applied, and only a three-point moving-average filter was used to suppress sensor-level noise. Step 1 in Figure 9d shows the SKT data obtained after applying the moving-average filter.

### 4.4. Feature Extraction

As the FMS score was collected at least every 30 s, handcrafted features were calculated using a 30-s window. For a session with an effective length of 1140 s, 38 aligned 30-s windows were generated. The FMS score for each window was mapped as the time-weighted mean of the FMS scores reported in the interval.

Most of the features from the HS-Set were adopted where possible. However, features that were impossible to calculate (e.g., the dataset did not include the pupil diameter), difficult to reproduce (e.g., three intrinsic mode functions of EDA), or had undisclosed calculation methods (e.g., the excitement feature from EEG) were excluded from the analysis. Furthermore, in the cases where features were classified under the same category in the HS-Set but had differing definitions or extraction procedures across studies or were overly granular, they were consolidated into representative features to reduce redundancy and ensure interpretative consistency. For example, the HR variability (HRV) feature group in PPG (SDSD, SDNN, RMSSD, pNN20, pNN50, etc.) was condensed to SDNN and RMSSD, the frequency-domain bands in EEG (simple frequency, standard frequency, and expanded frequency) were condensed to simple frequency and standard frequency, and the cardiac vagal tone (CVT) in PPG was replaced with the cardiac vagal index (CVI).

Each feature was classified as either a signal-wise feature (SwF), if it could be calculated as a time-series signal, or a window-derived feature (WdF), if it was summarized as a single scalar value within a given interval. For instance, the head movement features are SwFs, whereas the power ratio features of EEG are WdFs. Time- and frequency-domain templates, which are commonly applied to most sensors in the HS-Set, were used for SwFs. The mean, variance, kurtosis, skewness, sample entropy (embedding dimension *m* = 2, tolerance *r* = 0.2 × standard deviation (SD)) [137], and peak-to-peak amplitude were calculated in the time domain. In the frequency domain, the power spectral density (PSD) was obtained using Welch’s method. The DC component (0 Hz) was excluded, and any negative PSD values resulting from numerical noise were clipped to zero. Subsequently, the band power and Shannon entropy of the normalized PSD (hereafter referred to as PSD entropy) were calculated. However, if a window contained discontinuous segments owing to preprocessing, features were calculated for each segment and then integrated by averaging. The features extracted from each sensor are listed in Table 4. As an exception, body data were excluded from feature extraction because the tracking performance of the body joints of the Azure Kinect was unstable in the dynamic environment.

In the HS-Set, the only study that explicitly specified the low frequency (LF) and high frequency (HF) component bands for PPG was by Martin et al. [43], which used 0.04–0.15 Hz for the LF component and 0.15–0.4 Hz for the HF component. These are identical to the recommended standard bands for HRV analysis [139]. Accordingly, the HF component band for SwF extraction was set to the standard 0.15–0.4 Hz, because components below 0.1 Hz in the PPG data were suppressed during preprocessing, and the LF component band was set to 0.1–0.15 Hz. The time-domain SwFs for the LF and HF band signals were extracted by decomposition using an MNE band-pass filter, whereas the frequency-domain SwFs were extracted by decomposing the bands from the Welch PSD of the full-band signal (all subsequent band signal decompositions followed the same process). To stabilize the PSD entropy calculation for narrow bands, such as LF, the PSD was calculated with a fast Fourier transform (FFT) length of 8192 and a segment length of 20 s (n_per_segment = 2560) was used to account for the discontinuous data segments.

The WdFs for the PPG, such as HR, were calculated using the HeartPy package 1.2.7 in Python. Before the WdF calculation, a 0.66–3.33 Hz band-pass filter was applied to further suppress noise in the full-band signal. This range was set considering a typical heart rate of 40–200 bpm (≈ 0.66–3.33 Hz), a band also used by Martin et al. [43]. However, the package occasionally failed to compute SD1 and SD2. The missing SD1 values were calculated using the approximation formula SD1 ≈ RMSSD/√2 [140], and SD2 was calculated using the formula SD2^2^ ≈ 2 × SDNN^2^ − 1/2 × SDSD^2^ [141]. The CSI and CVI were calculated using the approximation formulae given by Toichi et al. [142]: CSI = L/T and CVI = log10(L × T), where T = 4 × SD1 and L = 4 × SD2.

While several studies involving the HS-Set used skin conductance level (SCL) and skin conductance response (SCR) components, none explicitly specified their frequency bands. Separately, Ishchenko and Shev’ev [143] and Greco et al. [144] reported that the band below 0.05 Hz corresponds to the SCL component and the 0.05–2 Hz band corresponds to the SCR component. Based on this, the SCL component band for extracting SwFs was set to 0–0.05 Hz, and the SCR component band was set to 0.05–2 Hz. In addition, an LF component with a 0.045–0.25 Hz band from Posada-Quintero et al. [138] was reported to be influenced by the sympathetic nervous system [144], and SwFs were also extracted from this component. Because the EDA data were collected using the same sensing equipment as the PPG data (Shimmer3 GSR+), they shared the same sampling rate (128 Hz) and the common issue of a narrow band for the SCL component, similar to the LF component of the PPG data. Accordingly, the frequency-domain SwF extraction process for the EDA data was identical to that for the PPG data.

Although the EEG studies in the HS-Set generally used similar bands to decompose the standard components, slight differences were observed, as shown in Table S9. The standard component bands for SwF extraction were decomposed using the most frequently used bands from the HS-Set studies. The upper cutoff frequency for the γ band was set to 50 Hz, chosen over the other frequently used value of 45 Hz, to encompass a broader range of information.

The time- and frequency-domain SwFs were integrated by averaging them for the global head (total channels), frontal lobe (F3, F4), central region (C3, C4), parietal lobe (P3, P4), and occipital lobe (O1, O2) (5 areas × 6 bands (whole + 5 standard bands) = 30 signals). Sample entropy, a time-frequency domain SwF, was calculated after downsampling to 125 Hz for computational convenience. The frequency-domain SwFs were extracted by decomposing the bands from the Welch-PSD of the full-band signal (1–50 Hz). As the EEG data were sampled at 500 Hz, the PSD was computed with an FFT length of 2048 (segment length = FFT length), which resulted in a frequency resolution of approximately 0.24 Hz/bin. Because the standard EEG bands are relatively wide (several Hz), this resolution can sufficiently distinguish the boundaries. Among the WdFs, the power ratio features were based on the seven features used by Sameri et al. [39]: β/α, (α + θ)/β, θ/α, θ/β, (α + θ)/(α + β), β/(α + θ), and Fθ/Pα. For the functional brain network (FBN), coherence and phase locking value (PLV) were used for each channel pair and were calculated for each of the six band signals (whole, 5 standard bands) (6 bands × _8_C_2_ × 2 metrics = 336 WdFs).

For the SKT data, only the SwFs from the full-band signal were used. As no separate band-pass filter was applied during preprocessing, the Welch-PSD was calculated with an FFT length of 20, corresponding to a sampling rate of 1 Hz × a 20-s segment length to account for discontinuous data. For the remaining sensor modalities, excluding PPG, EDA, EEG, and SKT, features were extracted only from the full-band signal because they had no sub-band components. The Welch-PSD for these signals was calculated using an FFT length corresponding to their sampling rate × 20 s, similar to the SKT data.

The data collected from the eye-tracking sensor of HoloLens 2 are limited to the eye position and gaze direction. However, the left/right eye data have null values or the same values as the central gaze (position and direction) when the eye is closed, and it is possible to perform a binary classification of the blink state. The closure signal, which classifies a closed state as 1 and an open state as 0, was calculated as

(1) $$\begin{array}{c} {CS}_{e}={\left\{1\left(isnull\left(P_{e}\left[k\right]\right)\;\vee {\left\|P_{e}\left[k\right]\;-P_{c}\left[k\right]\right\|}_{\infty }<10^{-3}\right)\right\}}_{k=1}^{T},\;e\in \left\{L,\;\;R\right\} \end{array}$$

where ***P*** is the eye position vector (*x*, *y*, *z*) in meters, L is the left eye, R is the right eye, *T* is the number of frames in the analysis interval, and ***P***_c_ is the origin of the HMD gaze. The threshold of 10^−3^ is the tolerance based on the L∞-norm, which is set conservatively to absorb slight rounding errors from floating-point operations and the potential for momentary omissions of actual eye closure periods due to the 30 Hz sampling.

Intermittent single-frame inversions were observed in each closure signal owing to sensor noise and sampling characteristics. To address this, isolated ones (0–1–0) were suppressed to 0, and isolated zeros (1–0–1) were suppressed to 1, and this process was repeated until convergence.

Spontaneous and reflex blinks in humans are fundamentally binocular, meaning that the left and right eyes are usually closed concurrently. Therefore, SwFs were extracted using a frame-by-frame logical AND signal (CS_L_∧CS_R_), reflecting the state in which both eyes were closed. Further, to account for minor asynchronous events, a frame-by-frame logical OR signal (CS_L_∨CS_R_), which allows only one eye to be closed, was also used for SwF extraction. The blink rate WdF was calculated by averaging the values computed for the left and right eyes.

In the HS-Set, studies that used the convergence distance mostly referred to the metric provided by the VIVE Pro Eye SDK. We defined the convergence distance as the minimum Euclidean distance between the closest points on the binocular gaze lines, which was calculated for frame *k* as follows:

(2) $$d_{conv}\left[k\right]=\{\begin{array}{cc} \frac{\left|\left(P_{L}-P_{R}\right)\;\;\cdot \;\;\left(D_{L}\left[k\right]\times D_{R}\left[k\right]\right)\right|}{{\left\|D_{L}\left[k\right]\times D_{R}\left[k\right]\right\|}_{2}} & if\;\;{\left\|D_{L}\left[k\right]\times D_{R}\left[k\right]\right\|}_{2}\;\geq 10^{-3}\\ NaN & otherwise \end{array}$$

where ***P*** is the eye position vector (*x*, *y*, *z*) in meters, ***D*** is the gaze direction vector (*x*, *y*, *z*), L is the left eye, and R is the right eye. The threshold of 10^−3^ is the tolerance for ‖***D***_L_ × ***D***_R_‖_2_, which is used to prevent the denominator from approaching zero when the left and right eye gazes are virtually parallel. The *d_conv_* data across all frames formed a time-series signal that was used for SwF extraction. Furthermore, as the likelihood of focus convergence failure increased with drowsiness or lack of concentration, the ratio of NaN values to the total number of frames was used as the WdF.

The gaze direction was collected as a unit vector in the head coordinate system (e.g., forward = (0, 0, 1)). Because the L2-norm of this vector is always 1, it has only two DOF, and its Cartesian components (*x*, *y*, *z*) are not independent, and thus the component-wise statistics are difficult to interpret. Therefore, the center gaze (*g*_x_, *g*_y_, *g*_z_) was converted to the spherical coordinates as gaze angles: *θ*_yaw_ = 180/π × arctan2(*g*_x_, *g*_z_) and *θ*_pitch_ = 180/π × arcsin(min{1, max{−1, *g*_y_}}). The SwFs for the gaze direction signal were extracted from the *θ*_yaw_ and *θ*_pitch_ signals. Berton et al. [80] mentioned the concept of eye-head coordination, the degree of alignment between the gaze and head direction. Since the gaze direction used in this feature extraction is relative to the head coordinate system, it can indirectly represent this concept (as the degree of alignment is expressed by the amount of variance). The gaze distance-to-center feature, which is a measure of the distance from a specific reference, can also be indirectly represented (as the distance from the center is expressed by the amount of variance).

The gaze velocity was calculated by extending the dot product angle between consecutive direction vectors from degrees per frame to degrees per second, and SwFs were extracted from the velocity signal. Referring to Salvucci and Goldberg [145] and Tobii’s identification by velocity threshold classification guidelines [146], samples in the gaze velocity signal exceeding 100°/s were classified as saccades and the rest as fixations. The ratios of saccade and fixation frames to the total number of frames were used as the WdFs. The path length WdF was defined as the cumulative sum of the degrees per frame and calculated using the process described above.

In the HS-Set, Lee et al. [76] converted the gaze distribution of 360° videos into a cubemap and corrected for distortion in the spherical projection by defining the visual entropy as the entropy of the normalized heatmap distribution on each face. In contrast, this study involved F-VIMS/S-VIMS environments with the collected gaze directions as unit vectors, making the direct application of the prior method inappropriate. Therefore, a 2D heatmap (64 × 64 bins, range = [−90°, 90°] × [−90°, 90°]) was constructed from the converted *θ*_yaw_ and *θ*_pitch_ signals, and its Shannon entropy was calculated and used as the heatmap entropy.

In the Head data, SwFs were extracted for each of the three components of position/acceleration (2 × (sway, heave, surge)) and rotation/angular velocity (2 × (pitch, yaw, roll)). Additionally, considering the use of geographic coordinates in the HS-Set, the rotation vectors were converted to direction vectors, and then spherical coordinate signals (*ϕ*_yaw_, *ϕ*_pitch_ signals) were calculated using the same procedure as the gaze direction. However, it is difficult to directly convert the head rotation into a direction vector because there is no central reference. Thus, the relative rotational difference between the frames was calculated based on the average rotation of the window, which was then applied to the forward direction vector (0, 0, 1), and the result was defined as the direction vector. SwFs were also extracted from the *ϕ*_yaw_ and *ϕ*_pitch_ signals and calculated using this process. The VOR represents the degree to which the gaze remains fixed in space as the head moves and is generally calculated as the ratio of the gaze velocity to head velocity. Therefore, the head velocity was calculated from the direction vector defined through the same procedure as the gaze velocity, and the mean of (gaze velocity/head velocity) over the window was used as the VOR WdF.

## 5. Results

To evaluate the factors contributing to MS across different stimulus conditions, the statistical analysis followed a structured roadmap. First, Section 5.1 profiles the participants’ demographic characteristics and baseline susceptibility to MS. Section 5.2 examines the main and interaction effects of MS stimulus type (within-subjects factor: CMS, VIMS, and Co-MS) and user characteristics (between-subjects factors: gender and susceptibility) on ΔSSQ using three-way mixed repeated measures analysis of variances (RM ANOVAs). In addition, because the display mode (F-VIMS vs. S-VIMS) is only applicable when visual stimuli are present, separate three-way ANOVAs were performed on the single-stimulus VIMS and Co-MS conditions to evaluate the main effects of the mode and its interactions with the user characteristics. Section 5.3 investigates the association between psychological immersion (Film IEQ) and MS symptoms, and Section 5.4 explores the correlations between multimodal human-signal features and subjective MSLs (ΔSSQ and FMS) at a nominal significance level. Finally, Section 5.5 evaluates the multivariable contributions of sensor domains and features to the FMS using an EBM and identifies practical lightweight combinations for actual vehicular environments.

### 5.1. Participant Demographics

The MS susceptibility of the participants was calculated using the MSSQ-Short percentile formulae [147]:

(3) $${MSSQ}_{percent}=Ax\;-B\;x^{2}\;-\;C\;x^{3}\;+\;D\;x^{4}$$

(4) $${MSSQ}_{score}=\frac{9\times \sum S_{\mathrm{child}}\;}{9-N_{\mathrm{child}}^{miss}}\;+\;\frac{9\times \sum S_{adult}\;}{9-N_{adult}^{miss}}$$

where *A* = 5.1160923, *B* = −0.055169904, *C* = 0.00067784495, *D* = 0.000010714752, *x* = MSSQ_score_, *S* is the score for each survey item, and *N*^miss^ is the number of types not experiencing MS. Although the actual susceptibility measurement used the MSSQ (0 = never, 1 = rarely, 2 = sometimes, 3 = frequently, 4 = always), the percentile conversion was performed using the MSSQ-Short scores from Golding [147] (0 = never, 1 = rarely, 2 = sometimes, 3 = frequently), and thus the scores were converted as follows: 4 points (always) → 3 points (frequently), with the remaining 0–3 points unchanged. Additionally, since the MSSQ-Short uses only sick/nausea scores, the vomiting scores were excluded from the calculation.

**Figure 10:**
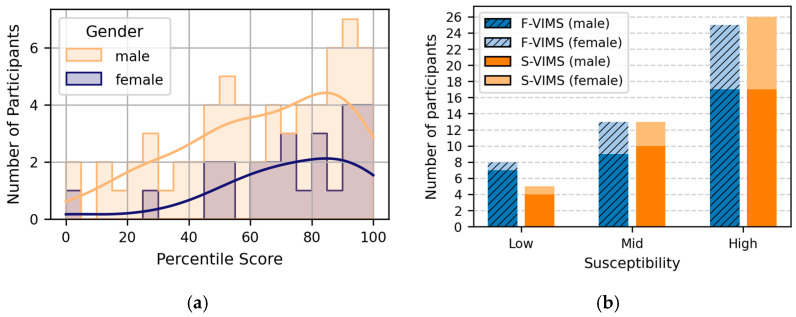
Frequency distribution of participants: (**a**) MSSQ percentile scores for the two genders, the lines represent the kernel density curves; (**b**) Distribution according to the VIMS type (F-VIMS, S-VIMS) for different MS susceptibilities and genders.

Participants were classified into tertile-based MS susceptibility groups based on their percentile scores: ≤33% = low susceptibility, >33 to ≤66% = moderate susceptibility, and >66% = high susceptibility. The distribution of the MSSQ scores of the participants is shown in Figure 10 and Table 5.

Figure 10b shows the frequency distribution of the participants by VIMS type according to their gender and susceptibility. Overall, the patterns for both the VIMS conditions were similar, with the high-susceptibility group having the most participants and the low-susceptibility group having the fewest. In addition, most female participants were concentrated in the moderate-to-high susceptibility group. Considering the relatively small number of participants in the low-susceptibility group, the low- and moderate-susceptibility groups were combined into a normal-susceptibility group for subsequent analysis.

### 5.2. Effects of the MS Types on the SSQ Scores

Table 6 shows the ΔSSQ for each MS type (CMS, VIMS, and Co-MS). Under single-stimulus conditions, the mean values of all ΔSSQs (T, N, O, and D) were the highest for CMS, followed by S-VIMS and F-VIMS. Furthermore, the mean ΔSSQ for Co-MS was generally higher than that for each single-stimulus condition. Among the subscales, there was a tendency for ΔSSQ_O_ to be relatively high and for ΔSSQ_N_ to be low, and the SDs indicated large individual differences.

A three-way mixed RM ANOVA was conducted to examine the effects of the type of MS, gender, and susceptibility level on ΔSSQ. As each participant was repeatedly exposed to the three conditions (CMS, VIMS, Co-MS), the within-subjects factor was the MS type, the dependent variables were the ΔSSQs (T, N, O, D), and the between-subjects factors were gender and susceptibility. Analyses were performed independently for each dependent variable using the afex package in R 4.3.3. The RM ANOVA in this package performs Mauchly’s sphericity test for reliability and applies the Greenhouse–Geisser correction if the assumption is violated. Type III sum of squares was used to control the potential imbalances and interaction effects. To reduce the impact of multiple comparisons, the test results were corrected using the Benjamini–Hochberg procedure with the family set for each dependent variable. The results of the three-way RM ANOVA for the S-VIMS participant group are shown in Table 7. None of the main or interaction effects were significant for ΔSSQ, and no trend (*p* < 0.1) was observed.

The results of the three-way mixed RM ANOVA performed on the F-VIMS group using the same procedure are shown in Table 8. Gender had significant effects on the ΔSSQ_T_ and the ΔSSQ_N_ and approached significance for the ΔSSQ_O_. Susceptibility did not have significant main effects on any SSQ subscales, but when combined with gender, it had a significant interaction effect on ΔSSQ_N_. The MS type had significant effects on all ΔSSQs (T, N, O, and D), and the interaction effect was significant only when combined with gender.

As the mixed RM ANOVA results differed between the S-VIMS and F-VIMS groups, a three-way ANOVA was conducted to examine the effect of the VIMS type on ΔSSQ. The analysis was performed on the single-stimulus VIMS data, excluding the CMS and Co-MS conditions from each VIMS type group, and thus, no repeated measurement factor was included. The dependent variables were the ΔSSQs (T, N, O, and D), and the between-subjects factors were the VIMS type, gender, and susceptibility. The test results were corrected using the same method as that for the mixed RM ANOVA. The results of the three-way ANOVA for the single-stimulus VIMS type are shown in Table 9. All main and interaction effects were nonsignificant, indicating that single-stimulus VIMS was not significantly affected by its type or user characteristics.

To confirm whether the VIMS type also had no effect in a combined-stimulus context, such as Co-MS, the conditions were changed, and another three-way ANOVA was performed. The analysis procedure was identical, but the target of the analysis was changed from the Co-MS condition to the combined-stimulus VIMS data. The results of the three-way ANOVA considering the combined-stimulus VIMS type are shown in Table 10. The interaction effect between gender and the VIMS type had a significant effect on ΔSSQ_N_ and approached significance for ΔSSQ_T_. The other main effects and interaction effects were not significant for any of the subscales. These results indicate that the influence of the VIMS type on Co-MS might be moderated by the user characteristics.

### 5.3. MS and Immersion

The subscales of the Film IEQ were calculated by a simple summation of item scores, and thus no separate score-conversion process was performed. Table 11 shows the Film IEQ subscale scores (IEQ scores) for the different VIMS types under single- and combined-stimulus conditions.

Significance was tested using a two-way mixed ANOVA. As each participant was repeatedly exposed to two conditions (VIMS and Co-MS), the within-subjects factor was set to the MS type. The dependent variables were the IEQ scores (Cap, Dis, Com, and Tra), and the between-subjects factor was the VIMS type (F-VIMS or S-VIMS). As the sample sizes were nearly balanced (the ratio between the VIMS and Co-MS conditions was the same, with 44 S-VIMS participants and 46 F-VIMS participants), a type II sum of squares was used. To reduce the impact of multiple comparisons, the test results were corrected using the Benjamini–Hochberg procedure with the family set for each dependent variable. The analysis was performed using the Pingouin package 0.5.5 in Python, the results of which are listed in Table 12. All effects on immersion were nonsignificant, indicating that the visual stimulus-inducing display modality and MS type had negligible effects on immersion.

**Figure 11:**
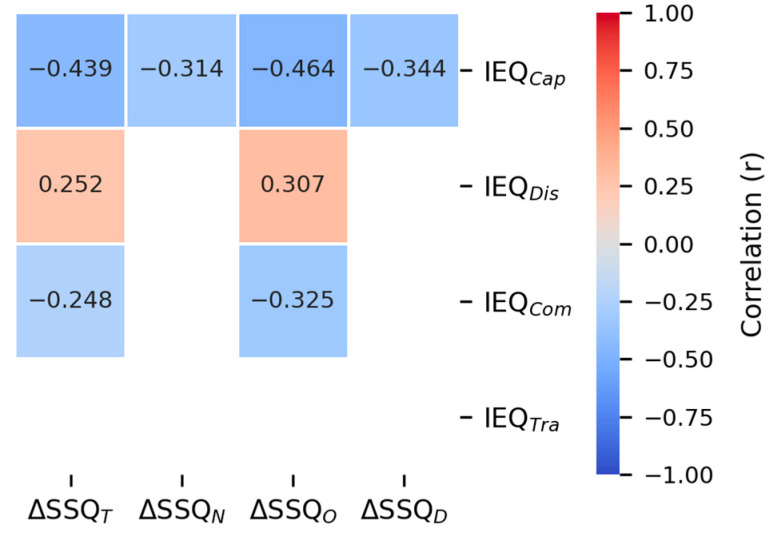
Repeated measures correlations between the IEQ scores and ΔSSQ. Blank cells indicate nonsignificant correlations (*α* = 0.05).

The association between immersion and symptom-specific MSL was examined using a repeated measures correlation (rmcorr) between the ΔSSQs (T, N, O, and D) and the IEQ scores (Cap, Dis, Com, and Tra), as shown in Figure 11. To reduce the impact of multiple comparisons, the test results were corrected using the Benjamini–Hochberg procedure, with the family set as all 16 correlation tests (Film IEQ subscales × SSQ subscales). IEQ_Cap_ exhibited significantly negative correlations with all ΔSSQs, and IEQ_Com_ exhibited significantly negative correlations with ΔSSQ_O_ and ΔSSQ_T_. In contrast, IEQ_Dis_ exhibited significantly positive correlations with the ΔSSQ_O_ and the ΔSSQ_T_, whereas IEQ_Tra_ was not significantly correlated with any of the ΔSSQs.

### 5.4. Correlation Between Human-Signal Sensing Data and MSL

This section presents the results of the correlation analyses performed between the two MSL measures (ΔSSQ and FMS) and the extracted features to explore nominally significant associations. The FMS was analyzed using features on a window-by-window basis, whereas ΔSSQ was analyzed using the mean features of the windows within a session. For the analysis, a baseline sample of 84 participants was used after excluding six participants due to the measurement errors from the acquisition system. Data from some of the participants were further excluded based on the signal quality of each sensor. EDA data were analyzed after excluding two participants with thin fingers, which caused unstable electrode-skin contact across all conditions (CMS, VIMS, and Co-MS) (*N* = 82). EEG data were analyzed after excluding four, three, and two participants in the CMS, VIMS, and Co-MS conditions, respectively, due to measurement errors and poor signal quality (*N* = 80/81/82).

Although various MS types were used in the experiment, core features with a common association could be identified regardless of the MS type. To examine this, the dataset was analyzed as a single set without partitioning by condition, and rmcorr was calculated to reflect the within-participant variance, considering that the same participant was repeatedly exposed to the three conditions (CMS, VIMS, and Co-MS). Furthermore, rmcorr also reflected the dependency arising from repeated measurements, as a maximum of 38 windows were generated per participant for the FMS. The two continuous variables were defined as pairs of each MSL metric (ΔSSQ_T_, ΔSSQ_N_, ΔSSQ_O_, ΔSSQ_D_, and FMS) and extracted features (ΔSSQ on a session basis, FMS on a window basis), and the within-subject correlation coefficient was calculated based on the participant identifiers. Each analysis included only cases with at least two observations per participant, and missing values were excluded pairwise.

**Figure 12:**
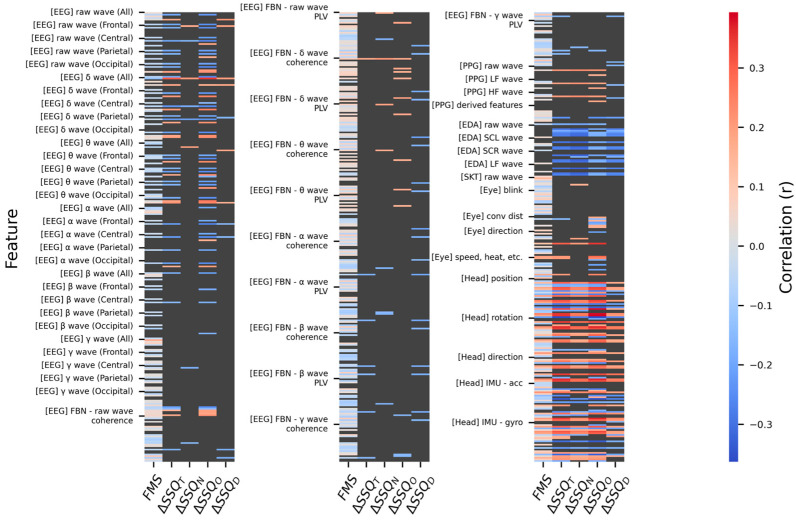
Repeated measures correlations between multimodal features and MSL (FMS, ΔSSQ). Black cells indicate nonsignificant correlations (*α* = 0.05).

As this correlation analysis was exploratory in nature, aiming to broadly identify potential candidates in a multivariate feature space, no correction for multiple comparisons was applied, which could have led to excessive Type II errors. Instead, statistical significance was assessed at *α* = 0.05, but a smallest effect size of interest (SESOI) of |*r*| > 0.25 (medium-large) was set as an additional criterion considering the nature of human-signal features [148]. Hereafter, a conservatively significant correlation refers to a correlation that satisfies both |*r*| > 0.25 and *p* < 0.05. Figure 12 shows the results of the rmcorr between the MSL metrics and the features, indicating that EDA and the Head features have a significant correlation with the overall MSL (*α* = 0.05).

While 10 features from the whole and standard band signals of the EEG had a conservatively significant correlation (*p* < 0.05 and |*r*| > 0.25), there were few significant items compared to the extracted features. To reduce candidate omission, the SESOI was relaxed to |*r*| > 0.20. The EEG features with medium-to-large significant correlations are listed in Table 13. Except for the γ-global-mean (band-area-feature), none of the β and γ features met the relaxed criteria. Considering that the significance of the whole band reflects its sub-bands, this indicates that the key bands are δ-α. Except for the parietal lobe, most correlations were confirmed only with the ΔSSQ_O_. This could be a result of the visual processing load in the occipital lobe and the visual/ocular control functions of the frontal lobe interacting with the central network, thereby reflecting visual fatigue and oculomotor load. Furthermore, parietal lobe features in the δ-θ signals showed a medium-to-large correlation with ΔSSQ_O_ and ΔSSQ_N_, which could be because the parietal lobe performs spatial cognition through vestibular information, thereby regulating eye-head coordination and postural stability. Among the main SwFs, sample and PSD entropy were negatively correlated with ΔSSQ, whereas kurtosis and skewness were positively correlated. This can be summarized as a common pattern in which the distribution sharpens, and asymmetry is enhanced as irregularity decreases. Among the WdFs, β/(α + θ) and (α + θ)/(α + β) had a medium-to-large significant correlation with ΔSSQ_O_. For ΔSSQ_N_, the δ-band F3-P3 coherence had a medium-sized correlation, and for ΔSSQ_D_, the δ-band O1-O2 PLV had a medium-sized correlation. However, the correlations of these WdFs and those of the frontal-lobe SwFs did not meet the conservative significance criterion, and thus they should be used as auxiliary indicators. In summary, the standard band signals showed significant correlations centered on oculomotor symptoms, with the key signal domains being frequency (δ–α) and space (frontal, central, occipital), and the parietal lobe served as an auxiliary signal.

In the PPG signals (whole and sub-band), no features with conservatively significant correlations were identified, and thus the SESOI was relaxed to |*r*| > 0.2 (medium). The PPG features with medium-sized significant correlations are listed in Table 14. Among the SwFs from the whole, LF, and HF band signals, only the variance was significantly correlated with all ΔSSQs (T, N, O, and D); these correlations were positive and medium-sized. This indicates that the variability of the pulse wave increased with the MSL. Among the WdFs, SD1/SD2 exhibited medium-sized negative correlations with ΔSSQ_T_, ΔSSQ_O_, and ΔSSQ_D_, implying that an increase in long-term variability (SD2) relative to short-term variability (SD1) is associated with an increase in oculomotor and disorientation severities. BR exhibited a medium-sized negative correlation with ΔSSQ_N_, indicating a tendency for the respiration rate to decrease with an increase in the severity of nausea. However, since the magnitude of all feature correlations was in the range of 0.20 ≤ |*r*| ≤ 0.25, PPG features are better interpreted as auxiliary indicators rather than primary.

In the EDA signals (whole and sub-band), 38 features with conservatively significant correlations were identified (Table 15). In contrast, for the SKT, no features met the criteria even when the SESOI was relaxed to |*r*| > 0.2 (medium). EDA exhibited conservatively significant correlations with ΔSSQs, except ΔSSQ_O_, across all bands (whole, SCL, SCR, LF). Unlike the other domains, certain features showed strong correlations (|*r*| > 0.3). In addition, the variance, peak-to-peak amplitude, and band power across all bands showed negative correlations with MSL, with the largest effect in ΔSSQ_N_ (|*r*| ≈ 0.30–0.33). This indicates that EDA can be effectively utilized in environments with physical stimuli such as CMS and Co-MS. Overall, all bands showed similar patterns (symptoms, effect sizes, and features), but the whole-band signal showed conservatively significant correlations in skewness, unlike the sub-bands. The significant appearance of skewness only in the whole band is because the high-amplitude peak characteristics were dispersed through the band decomposition process, alleviating asymmetry.

For the eye sensor domain, only three SwFs had conservatively significant correlations: PSD entropy in the yaw direction with ΔSSQ_T_ (*r* = 0.304, *p* = 0.002) and ΔSSQ_O_ (*r* = 0.304, *p* = 0.002), and PSD entropy in the pitch direction with ΔSSQ_O_ (*r* = 0.283, *p* = 0.003). This indicates that as oculomotor symptoms intensify, the band power of eye movements tends to disperse across multiple frequencies rather than concentrating on a few dominant rhythms. However, as the number of significant items in the eye-tracking data was too small relative to the number of extracted features, the SESOI was relaxed to |*r*| > 0.20, to reduce candidate omission, and features with a significant correlation under this criterion were additionally identified. The eye-tracking features with medium-to-large significant correlations are listed in Table 16. Except for ΔSSQ_T_, most features had a medium-to-large significant correlation with ΔSSQ_O_. The PSD entropy of the yaw and pitch directions had consistent positive correlations with ΔSSQ_T_, ΔSSQ_O_, and ΔSSQ_N_. Most velocity SwFs were positively correlated with ΔSSQ_T_ and ΔSSQ_O_, except for skewness and PSD entropy, which were negatively correlated. In addition, the cumulative angle WdF was confirmed to have a positive correlation with ΔSSQ_O_, and the kurtosis of the convergence distance had a negative correlation. In summary, as the oculomotor symptoms intensified, the gaze direction dispersed across multiple frequencies, leading to an increase in trajectory changes and movement distance. In contrast, the velocity spectrum and distribution became regularized, reducing the number of extreme peaks, representing a dual pattern. This was complementary to the pattern observed in the frontal and occipital lobes of the EEG (entropy↓, kurtosis↑) and was interpreted as the result of the burden on vision-oculomotion-visual processing being reflected in the ΔSSQ_O_. However, because these features had medium-sized significant correlations under relaxed conditions, they were interpreted as auxiliary indicators.

In the head sensor domain, 94 features were identified to have conservatively significant correlations. To avoid overinterpretation due to redundant features, the SESOI was raised to |*r*| > 0.3 (large). The number of head-tracking features with a large-sized significant correlation was 41, as shown in Table 17. Features with a large-sized significant correlation tended to concentrate on ΔSSQ_O_ and ΔSSQ_T_. Overall, the amplitude/energy features (variance, ptp, and band power) had strong positive correlations with ΔSSQ_O_ and ΔSSQ_T_. In terms of feature metrics, the amplitude/energy features of the pitch/surge exhibited the most consistent positive correlation, and similar tendencies were partially observed for heave and sway. For acceleration/angular velocity, these metrics also showed a predominantly positive correlation with ΔSSQ_O/T_. This indicates that as oculomotor symptoms intensified, the amplitude and energy of head movements tended to increase and were centered on the pitch and surge axes. In contrast, irregularity features (kurtosis and sample/PSD entropy) showed strong negative correlations with ΔSSQ_O_ and ΔSSQ_T_. This implies that as the oculomotor symptoms intensified, the position and rotation tended to converge to a specific pattern. Furthermore, the head sensor domain had 94 features with conservatively significant correlations (41 even when retaining only large criteria |*r*| > 0.3), a richer extraction than other domains, and a consistent correlation structure across multiple features. This supports the practicality of head-tracking features as a key, low-burden, and lightweight indicator alongside EDA.

For the FMS, no features from any sensor domain exhibited conservatively significant correlations. Only when the SESOI was relaxed to |*r*| > 0.2 (medium), features with a medium-sized significant correlation were identified (Table 18). All FMS-related features with a medium-sized significant correlation were the head-tracking features, and their correlation structure was consistent with the correlation between these features and ΔSSQ. However, considering that ΔSSQ_T_ was influenced by ΔSSQ_O_, it was difficult to estimate the severity of nausea and oculomotor symptoms in real time using human-signal features that had common correlations across various MS environments.

**Figure 13:**
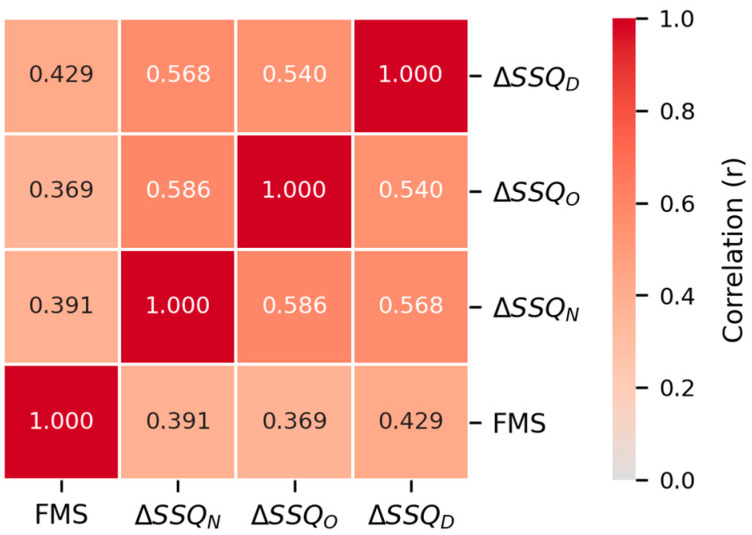
Repeated measures correlations within MSL indices (ΔSSQ_N_, ΔSSQ_O_, ΔSSQ_D_, and FMS). All cells indicate significant correlations (*p* < 0.001).

To confirm whether FMS concentrated on ΔSSQ_O_ in these results, rmcorr was used for examination. To reduce the impact of multiple comparisons, test results were corrected using the Benjamini–Hochberg procedure, with families set as six correlation tests being pairwise combinations of four MSL measures (ΔSSQ_T_, ΔSSQ_N_, ΔSSQ_O_, and FMS). As shown in Figure 13, FMS exhibited significant positive correlations with all ΔSSQs, indicating that FMS is not limited to ΔSSQ_O_.

### 5.5. Relative Contributions of Multimodal Features to MSL

Statistical analyses effectively identify significant individual factors; however, MSL can involve complex nonlinearity and feature interactions among the multimodal signals. To investigate these complex dynamics and determine the relative importance of each sensor domain, a contribution analysis was performed using an ML approach. In this approach, demographic variables were included as input features because they were confirmed to have significant main or interaction effects (Section 5.2).

An EBM was used as the ML model to ensure interpretability. EBM, which combines a generalized additive model with second-order interactions, can transparently and quantitatively explain nonlinear relationships by estimating the effect curve of each feature and pairwise interaction as separate additive terms. For the regression target, the continuous FMS score was directly employed instead of the session-based ΔSSQ. Although the correlation coefficients between the FMS and SSQ subscales were moderate, they were statistically significant across all symptom categories, indicating that the FMS serves as a valid representative indicator of diverse MS symptoms.

**Figure 14:**
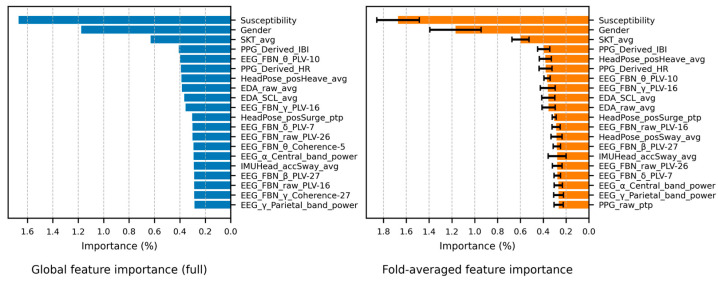
Top-20 global feature importance values (%) from the EBM models. The black error bars in the right panel represent the standard deviation across the 10-fold cross-validation.

For post hoc explanatory attribution, the EBM was fitted to the entire dataset to obtain global feature importances (seed = 42, interaction = 0.9, validation ratio = 0.2, goodness of fit (R^2^) = 0.919, Pearson linear correlation coefficient (PLCC) = 0.961, and Spearman’s rank correlation coefficient (SRCC) = 0.877). To rigorously assess the robustness of these feature contributions and ensure that they were not merely artifacts of the dataset, the analysis was supplemented with participant-wise cross-validation. Figure 14 shows the top 20 most important features derived from the full dataset model alongside the mean feature importances maintained across the 10-fold cross-validation.

Among the multimodal features, demographic variables (susceptibility and gender) and skin temperature (SKT_avg) consistently ranked among the strongest multivariable contributors to MSL. For physiological responses, cardiovascular metrics (PPG_Derived_IBI and HR) and electrodermal indices (EDA_raw_avg and SCL_avg) repeatedly appeared in the upper ranks, indicating their stable contributions. Regarding head motion, although the univariate correlation analysis (Section 5.4) highlighted broad linear associations between head-movement features and MS, the EBM emphasized only a small set of representative translational kinematics (e.g., HeadPose_posHeave_avg and HeadPose_posSurge_ptp) among the top-ranked predictors. The most distinct pattern relative to the univariate results was observed for EEG: while individual EEG features showed weaker linear associations, the EBM consistently identified multiple FBN connectivity features (e.g., PLV and coherence-related indices across several frequency bands) as important nonlinear contributors to MSL. Conversely, no gaze features were included in the top-20 multivariable contributors.

**Figure 15:**
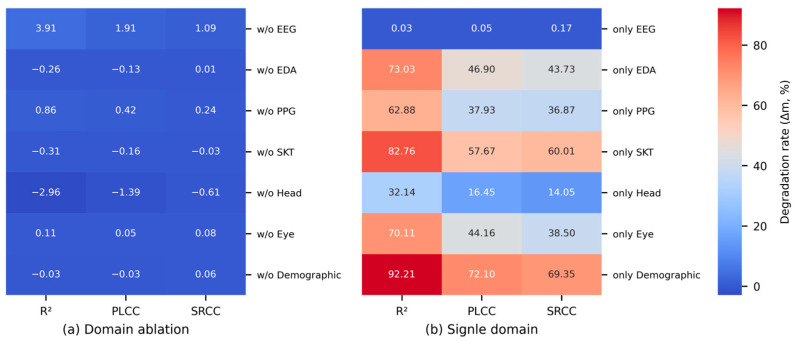
Relative degradation in explanatory power for the sensor domains (w/o = without).

The global importance of the model was widely distributed across many features, making it difficult to intuitively identify the sensor domains that contributed the most. Furthermore, owing to the imbalance in the number of features across the domains, the possibility that the model over-relied on a specific domain could not be ruled out. Therefore, an ablation study was conducted by refitting the model after removing all features from each sensor domain while keeping the dataset and hyperparameters the same. The change in explanatory power was defined as the relative degradation rate compared to the baseline model as follows:

(5) $$\begin{array}{c} \Delta m\left(\%\right)=\frac{m_{full}-m_{abl}}{m_{full}}\times 100,\;m\in \left\{R^{2},\;\;PLCC,\;\;\;SRCC\right\} \end{array}$$

where Δ*m* > 0 indicates a degradation in explanatory power (decrease), and Δ*m* < 0 indicates that the model fit improved upon removal. To complement the ablation analysis and address the imbalance in feature counts across domains, single-domain models were also refitted. Figure 15 summarizes the relative degradation in explanatory power when each domain was excluded (w/o) and when only a single domain was used.

In the domain ablation analysis (Figure 15a), the EEG-ablated model exhibited the largest degradation across all metrics (R^2^, PLCC, and SRCC). However, the magnitude of this reduction was relatively modest, not exceeding 5% (e.g., 3.91% for R^2^). Furthermore, the ablation of the remaining domains (EDA, PPG, SKT, Head, Eye, and Demographic) resulted in marginal decreases of less than 1% or even slight improvements in the model fit. Notably, the head-ablated model showed a clear improvement in model fit, indicated by a negative degradation rate of −2.96% for R^2^ relative to the full model under the same dataset and hyperparameter settings. Although EEG ablation produced the largest degradation among the domains, it should be noted that the EEG features accounted for 583 out of 828 total features (≈ 70.4%), representing a very high proportion. As a complementary analysis, a single-domain evaluation was conducted (Figure 15b) to examine this imbalance and highlight domain contributions without inter-domain redundancy.

The single-domain models provided a domain-wise comparison of explanatory power. The EEG-only model exhibited an exceptionally low degradation rate (0.03% in R^2^), maintaining an R ^2^ almost equivalent to that of the full model. The head-only model recorded the second-lowest degradation rate (32.14% in R^2^). Among the physiological signals, the PPG-only model showed the next lowest degradation (62.88% in R^2^). The other single-domain models (Eye, EDA, SKT, and Demographic) exhibited substantial degradation in model fit (over 70% in R^2^). Although the EDA- and Eye-only models retained partial correlations, with 38.50–46.90% drops in PLCC and SRCC, these substantial losses still quantify their limited standalone modeling capacity.

**Figure 16:**
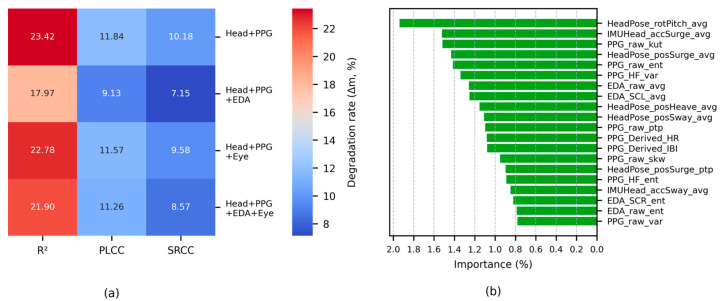
Evaluation of lightweight multimodal combinations: (**a**) Relative degradation rates for the four sensor combinations excluding EEG. (**b**) Top-20 global feature importance values (%) for the optimal combination (Head + PPG + EDA).

Given the practical constraints of scalp EEG in actual vehicular environments, lightweight sensor combinations excluding EEG were further evaluated. Based on the domain hierarchy observed in the single-domain evaluation, the Head and PPG were selected as the common baseline modalities. The EDA and Eye domains were conditionally added, resulting in four combinations: Head + PPG, Head + PPG + EDA, Head + PPG + Eye, and Head + PPG + EDA + Eye. As shown in Figure 16a, the Head + PPG + EDA combination exhibited the lowest degradation in model fit across all metrics (17.97% for R^2^, 9.13% for PLCC, and 7.15% for SRCC), demonstrating the optimal explanatory capacity among the lightweight models.

The global feature importances for the optimal Head + PPG + EDA combination are presented in Figure 16b. Consistent with the findings from the full multimodal evaluation, head kinematic features maintained the highest ranks, with variables such as average pitch rotation (HeadPose_rotPitch_avg) and surge acceleration (IMUHead_accSurge_avg) serving as the primary explanatory indicators. Furthermore, metrics from the PPG and EDA domains, including raw signal statistics (e.g., PPG_raw_kut and EDA_raw_avg) and derived physiological indices (e.g., PPG_HF_var and EDA_SCL_avg), constituted the remaining top ranks. Overall, head kinematics remained prominent in the lightweight setting, whereas cardiovascular and electrodermal features contributed additional explanatory information.

## 6. Discussion

MSL was quantitatively examined from a multimodal human-signal perspective under single and composite stimuli in the context of autonomous driving. Considering that the MS quantification studies in the HS-Set were biased towards VR-based VIMS, this study complements the gap in MS quantification research by providing a unified examination of physical stimuli that simulate driving, visual stimuli, and their combination, while also covering see-through HMD and simulator environments.

### 6.1. Investigation of User Characteristics and Immersion Effects Across MS Types

The absence of significant MSL differences across MS types and user characteristics in the S-VIMS group suggests that, from the perspective of the sensory conflict theory, stimulus variables not treated as independent factors in this study (e.g., stimulus intensity such as optical flow and physical movement of the simulator itself) likely exerted a more dominant influence on MSL than demographic and MS-type variables.

Conversely, the F-VIMS group exhibited significant MSL variations across MS types, generally following an ascending order of VIMS < CMS < Co-MS. The exceptionally low MSL under the single visual stimulus condition may stem directly from the limited field of view (FOV) of the consumer-grade tablet, which likely weakened vection. The subsequent peak in MSL during Co-MS suggests that the combination of a narrowed visual field caused by content immersion and a tilted vestibular axis from a downward posture (neck flexion) amplified the visual-vestibular sensory conflict, a phenomenon absent in the open visual field of S-VIMS. This interpretation is further corroborated by the high predictive importance of the amplitude and energy features of the head domain. Furthermore, the unique deviation for oculomotor symptoms—where CMS induced higher severity than Co-MS—might indicate that physical motion destabilized gaze fixation and tracking, while repetitive and monotonous visual stimuli hindered immersion and exacerbated visual fatigue.

Despite the use of different display technologies (F-VIMS vs. S-VIMS), the display type itself does not appear to be a primary determinant of MSL quantification under single-stimulus conditions. However, the emergence of significant gender effects (a gender effect in F-VIMS and a gender × VIMS type interaction in Co-MS) specifically impacting nausea suggests that gender acts not as a constant main effect but rather as a conditional amplifier. The absence of this effect in S-VIMS implies that the specific sensory composition, such as display type, posture, and FOV, may modulate the expression threshold of gender-related susceptibilities. Consequently, the design of personalized MS-mitigation strategies requires the distinction of these interaction-driven indicators.

Regarding psychological immersion, the lack of significant effects from either the display modality or the MS type on the Film IEQ subscales implies that user immersion is not directly governed by the technical specifications of the stimulus or the presence of composite stimuli. Consistent with prior research, this finding reaffirms that immersion relies more heavily on content quality and individual cognitive characteristics.

The observed negative correlations of both captivation and comprehension with MS symptoms support the capacity theory, indicating that deep cognitive engagement and higher predictability of the stimulus reduce the resources available to process visuo-vestibular conflicts, thereby facilitating adaptation and alleviating MS. Conversely, real-world dissociation exhibited a positive correlation with oculomotor and total MS symptoms but notably lacked an association with disorientation. This divergence highlights the intrinsic difference between the two concepts: real-world dissociation captures the subjective psychological perception of detachment, whereas disorientation reflects a physiological state of spatial confusion. Thus, despite conceptual similarities, the underlying mechanisms differ, demonstrating that psychological immersion experiences and physiological MS symptoms do not invariably maintain a one-to-one correspondence.

Finally, despite the use of subjective camera techniques, the transportation metric—indicating the sensation of being mentally transported—did not significantly correlate with MSL. This lack of association may stem from the inherent mismatches in the Co-MS condition, where the conflict between the simulator’s physical motion and the film’s visual motion disrupted the psychological experience of entering the narrative space. Consequently, narrative immersion appears to be fundamentally constrained by sensory conflict in composite-stimulus environments.

### 6.2. Quantitative Approaches to MS Based on Human Signals

Each human-signal domain exhibited unique characteristics based on the correlation between the extracted features and MSL. In the EEG domain, decreases in entropy and increases in kurtosis and skewness in the δ-α bands showed significant correlations only with oculomotor symptoms, reflecting the phenomenon of brainwave patterns converging on a specific rhythm. The overall correlation, with the oculomotor symptoms observed broadly across the fronto-central-occipital regions, and the partial correlation, with nausea observed in the parietal lobe, are consistent with the perspective of the sensory conflict theory that MS is fundamentally an issue of visuo-vestibular sensory integration. The finding that the FBN features, of which only three had a medium-sized correlation, were numerous in the top ranks of the EBM suggested that nonlinear feature interactions in EEG were more important for MS prediction than simple linear correlations. This implies that complex connectivity patterns between brain networks might reflect MSL changes more sensitively than independent contributions of individual features.

The EDA domain had features with consistently negative correlations across all bands, and features related to nausea exhibited strong correlations (|r| ≥ 0.3). Considering that Kenward et al. [110] and Wickham [111] reported that nausea is a higher-level cognitive activity than vomiting, this strong correlation suggested that EDA was able to sensitively capture the complex physiological and cognitive responses to nausea symptoms. In contrast, the PPG features showed broad correlations with all symptoms, but were only of medium size, making them more suitable as auxiliary indicators rather than core indicators. The increase in the variance (regardless of the band) exhibited medium-sized significant positive correlations with an increase in MSL, whereas SD1/SD2 exhibited medium-sized significant negative correlations, reflecting an increase in the pulse wave variability and a relative decrease in the parasympathetic tone, respectively. In other words, PPG appears to have captured a general, but relatively insensitive, cardiovascular response to MS.

The Eye and Head domain features exhibited complementary characteristics, capturing different aspects of the oculomotor symptoms. The head domain features showed the richest and most consistent correlation structures, with amplitude/energy features in the pitch and surge axes showing strong positive correlations, which reflected an increase in compensatory head movements in response to anteroposterior visual stimuli. In particular, the strong negative correlation of irregularity features (e.g., entropy and kurtosis) indicated a tendency for head movements to converge to a specific pattern as the oculomotor symptoms intensified, which can be interpreted as an adaptive response of the vestibular system. In the Eye domain, most features with medium-to-large correlations were in the frequency domain (PSD entropy), with positive correlations observed between nausea and oculomotor symptoms. This implies that as the MSL increased, eye movements became more irregular and dispersed across multiple frequencies. However, the VOR (gaze velocity/head velocity) used as an eye feature did not have a medium-to-large significant correlation, suggesting that the traditional VOR concept alone was insufficient to fully explain the complex visuo-vestibular interactions in MS. This result shows that the core mechanism of oculomotor symptoms is a complex response due to visual attention dispersion and visuo-vestibular conflict, rather than a simple VOR gain. Overall, considering that previous studies mainly dealt with eye data in the time domain, this result demonstrates the need to consider the frequency domain as well.

The significant positive correlation between the FMS and all ΔSSQ subsymptom scales supports the validity of the FMS as a window-level MSL indicator that encompasses various MS symptoms. This means that the FMS can reflect the overall MSL in a balanced manner without being biased towards a specific symptom. However, the relatively small correlations between the FMS and individual human-signal features reveal the inherent difficulty of real-time MSL quantification. This could be due to temporal delays or complex nonlinear relationships between the momentary subjective experience captured by the FMS and the physiological changes reflected by the human signals.

#### Multimodal Contribution Patterns Revealed by Interpretable Modeling

The interpretable modeling results complement the inferential and correlation analyses by characterizing the multivariable contribution patterns to MSL when nonlinearities and feature interactions are present. A notable observation was that global importance was not concentrated in a small set of predictors; instead, relatively small per-feature contributions were distributed across several multimodal variables. Together with the consistently high ranks of demographic variables and SKT-related indices, this pattern supports the view that MSL is expressed as an integrated response spanning multiple physiological and behavioral channels, rather than being determined by a few highly dominant factors captured by univariate associations alone.

Domain-level ablation and single-domain evaluations provided complementary perspectives on how explanatory capacity was distributed across modalities under substantial feature-count imbalances. In particular, EEG contributed strongly to the domain-wise comparisons; however, the overall ablation magnitudes were modest, and the model fit occasionally improved when specific domains were removed. Such behavior is consistent with substantial redundancy and shared information across modalities in a multivariable setting, where the presence of one domain can partially overlap with the nonlinear information represented by others. Accordingly, it is highlighted that domain relevance in this framework is more appropriately interpreted in terms of incremental changes in model fit, rather than being directly inferred from the number of significant univariate correlations.

From a practical standpoint, the strong contribution of EEG must be weighed against the constraints of in-vehicle deployment. Given the acceptability and setup burden of scalp EEG, EEG-excluded lightweight combinations were evaluated. Among the tested candidates, the Head + PPG + EDA configuration exhibited the most favorable model fit, and its global importance pattern was distributed across multiple features. Head kinematics consistently ranked the highest, while peripheral autonomic indices from PPG and EDA provided additional explanatory contributions, indicating that substantial MSL-related variation can be captured by combining motion-derived behavioral cues with peripheral physiological responses under constrained sensing conditions.

Finally, the limited degradation observed when demographic variables were excluded suggests that a meaningful portion of MSL-related patterns can be characterized using human signals alone, which has practical value for privacy-preserving monitoring when personal information is unavailable. Nevertheless, further validation remains necessary under operational driving conditions, where motion artifacts, sensor stability, and environmental noise may alter the signal quality and relative contribution patterns across MS-inducing factors.

### 6.3. Limitations

The participants for this study consisted of young adults in their early 20s from a single institution (male/female = 64:26). Therefore, the results of this experiment might be limited to young adults. Susceptibility was also managed as a two-level variable, normal (combining low and moderate) and high, based on MSSQ-Short. This dichotomization could have led to a conservative estimation of interaction effects (e.g., gender × susceptibility and display × susceptibility) and might not fully explain the heterogeneity within the normal group (low versus moderate). In addition, because detailed histories regarding the participants’ current pharmacological status and prior experience with see-through HMDs were not collected, this study has limitations in fully accounting for their potential effects on the results. Certain medications may alter baseline physiological signals, and prior HMD familiarity can lead to habituation, which naturally affects the VIMS threshold.

Although Co-MS was intentionally designed to induce visuo-vestibular conflict, it differs from the composite stimuli of actual road environments (e.g., road surface irregularities, cross slopes, traffic situations, and sudden lane changes). The use of heave/roll in the motion platform was limited, and the classic washout filter could not perfectly replicate the low-frequency vibration components of actual vehicles.

The VIMS stimulus was induced in two subjective camera films (found footage). However, for ecological validity, covariates dependent on directorial choices, such as scene transitions and luminance, were not held constant or removed. Therefore, the estimated effects are closer to the net effect under realistic conditions where content components coexist. However, there are limitations in isolating the contribution of each factor. In addition, F-VIMS (tablet) and S-VIMS (see-through HMD) have structural differences (e.g., FOV, luminance, and viewing distance), which limit a pure comparison of the display effects.

The session order was fixed (CMS → VIMS → Co-MS), which may have introduced carryover/late-session effects (e.g., fatigue, habituation/adaptation, or sensitization). Because Co-MS was always administered last, some portion of the observed Co-MS responses may reflect the accumulated session effects rather than the combined stimulation per se. To mitigate this, the rest was set to 12 min based on prior evidence of EEG recovery following MS exposure [88], and participants could stop at any time if the symptoms became severe. Nevertheless, since the session-type order was not randomized, residual order confounding cannot be fully ruled out, and Co-MS findings should be interpreted cautiously.

Subjective MSL was measured using SSQ (pre/post) and FMS (at 30-s intervals), making it difficult to perfectly capture sudden symptoms. Repeated responses to the FMS itself might have also induced attentional distraction. Although the time axis of the human signals was corrected through synchronization and interpolation, the possibility of slight time warping owing to multithreaded collection and differing sampling rates (30–500 Hz) remains.

The eight-channel resolution of the EEG constrained the precision of interpretation, and the 30 Hz measurement rate of the HoloLens 2 eye tracking resulted in missing data. In addition, PPG was used instead of ECG, but had low precision and artifact resistance, and EDA experienced some data loss owing to unstable contact for participants with thin fingers. Further, some features were indirectly derived indicators, such as BR, which was derived from the PPG instead of a dedicated RSP sensor. Continuous BP monitoring was also omitted to avoid physical distraction, although the extracted PPG features partially compensated for this absence by indirectly capturing cardiovascular dynamics.

While the implemented acquisition system enabled parallel collection across varying sampling rates, minor timing misalignments occurred owing to OS time resolution and thread scheduling limits. Specifically, the actual number of samples within a 1-s interval deviated minimally (e.g., by approximately ±1 chunk) from the expected grid. Although we applied piecewise cubic Hermite interpolation to tightly preserve the original signal morphology and minimize distortion during this time-axis correction, this study did not quantitatively profile the exact proportion of repaired or interpolated segments per sensor modality. Consequently, we cannot completely rule out the possibility that these corrected segments, however minimal, may have partially influenced specific feature calculations. Multiple comparison correction was applied only to the main hypothesis tests (ANOVA, rmcorr), whereas the extensive feature-symptom correlation analysis was treated as exploratory and reported by focusing on the effect sizes instead of adjustments.

The EBM utilized in this study was primarily designed for post hoc explanatory modeling, specifically to assess the relative multivariable contributions of multimodal features, rather than to construct a generalized, real-time prediction system for unseen passengers. Because the modeling objective was targeted at interpretability rather than maximizing predictive performance, extensive comparative benchmarking against predictive baselines was not performed. Furthermore, while participant-wise cross-validation was employed to ensure the robustness of the derived feature importance rankings, the current model focuses on evaluating the concurrent symptom state rather than forecasting future MS onset. Consequently, the reported feature contributions should be strictly interpreted as an internal attribution of multimodal factors within the current sample, as the structural dependencies of the explanatory framework inherently constrain its direct application as a generalized, real-time predictive metric for novel users.

In addition, the multivariable contribution analysis faced a structural limitation due to the substantial imbalance in the number of extracted features across sensor domains, with EEG alone accounting for approximately 70% of the total feature set. Although a single-domain evaluation was explicitly conducted to partially mitigate inter-domain redundancy and isolate individual domain effects, the sheer volume of EEG features intrinsically provided this domain with greater model capacity. Consequently, the observed superiority of EEG in preserving model fit may be partially amplified by its higher degrees of freedom, rather than being solely driven by its inherent informative value.

## 7. Conclusions

In this work, an integrated framework was designed for CMS-VIMS-Co-MS in the context of autonomous driving, and multifaceted analyses of MSL were performed by simultaneously collecting data from the EEG, EDA, PPG, Eye, Head, and SKT domains in two environments (F-VIMS/S-VIMS) (*N* = 90). Unlike previous studies that were limited to a single device or type, in this study, the display (tablet/see-through HMD), stimulus type, user characteristics (gender and susceptibility), and immersion were compared within a single framework.

The main results obtained from this study are as follows: (i) In the S-VIMS group, there were no significant differences in ΔSSQ according to the MS type, gender, or susceptibility. (ii) In the F-VIMS group, the main effect of the MS type was significant for all sub-symptom scales, with the general order being VIMS < CMS < Co-MS, with the exception of CMS > Co-MS, which was observed only for oculomotor symptoms. (iii) For single-stimulus VIMS, there was no ΔSSQ difference between F-VIMS and S-VIMS, and for Co-MS, the gender × VIMS type interaction was significant for nausea. (iv) IEQ showed no significant difference between F-VIMS and S-VIMS. In rmcorr, patterns of IEQ_cap_ (negative with all ΔSSQ), IEQ_com_ (negative with ΔSSQ_O_ and ΔSSQ_T_), IEQ_dis_ (positive with ΔSSQ_O_ and ΔSSQ_T_), and IEQ_tra_ (non-significant) were confirmed. (v) FMS was positively correlated with all ΔSSQs.

The correlations between human-signal features and MSL revealed distinct patterns across domains. In the EEG domain, a decrease in the entropy and an increase in the skewness and kurtosis in the δ-α bands were primarily linked to oculomotor symptoms. The PPG domain exhibited medium-sized correlations across all symptoms, acting more as an auxiliary indicator than a core indicator. The EDA domain frequently exhibited negative correlations of |*r*| ≥ 0.3, particularly with nausea. In the Head domain, the amplitude/energy features of the pitch/surge showed large positive correlations with oculomotor symptoms, whereas the irregularity features (e.g., entropy) showed negative correlations. In the Eye domain, PSD entropy was positively correlated with nausea/oculomotor symptoms, whereas traditional VOR did not have medium-to-large correlations.

The interpretable modeling based on the EBM demonstrated that multivariable contributions to the FMS were distributed across several multimodal features rather than concentrated on a few dominant predictors. The domain ablation and single-domain evaluations confirmed that EEG was the most important explanatory domain, whereas other single modalities (such as Eye, EDA, and SKT) showed substantially lower standalone fit than EEG, Head, and PPG. Nevertheless, the evaluation of lightweight sensor combinations identified the Head + PPG + EDA configuration as the optimal alternative, exhibiting minimal degradation in explanatory power. Notably, this confirmed the feasibility of characterizing MSL-related patterns using only human signals, even when demographic features were excluded.

The main contributions of this study are as follows: (i) An experimental framework was established to analyze CMS-VIMS-Co-MS within a single framework in the context of autonomous driving, thus complementing the limitations of previous fragmented studies and extending the research scope to next-generation displays. (ii) Through a systematic literature review of the last decade, the usage trends and classification system of features according to the human-signal domain were comprehensively organized, thus providing a reference for enhancing the methodological consistency and reproducibility of future MS-related human-signal studies. (iii) Through multimodal human-signal and MSL correlation analyses, significant features and symptom-specific patterns were derived for each sensor domain. (iv) Through EBM-based domain ablation/single-domain analysis, the core explanatory domain (EEG) and the relative multivariable contributions of multimodal features to the FMS were identified, and the effectiveness of lightweight sensor combinations (Head + PPG + EDA) was examined, thereby demonstrating the practical feasibility of few-sensor-based MSL assessment.

Future research will aim to perform nonlinear quantification of MSL based on DL by combining the stimulus intensity (six-DOF movement of the motion platform and first-person frame of the HMD) with lightweight domains (head, EDA, and PPG-based). Furthermore, despite the correlation structure between the FMS and SSQ, different correlational features were identified for nausea, oculomotor symptoms, and disorientation. Therefore, we intend to explore the latent patterns of MSL from human signals. Subsequently, an expansion to a real-world vehicle environment is planned to examine the generalization and operational feasibility of the MSL prediction model.

## Figures and Tables

**Table 1:** Frequencies of human-signal sensor domains in the HS-Set (not mutually exclusive).

Sensor Domain	Signal Type	Count	Included Studies
Electrocardiogram and Photoplethysmogram ^1^	physiological	35	[11,27,28,29,33,34,35,36,37,38,39,40,41,42,43,44,45,46,47,48,49,50,51,52,53,54,55,56,57,58,59,60,61,62,63]
Electrodermal activity	physiological	29	[11,27,32,36,37,38,39,40,41,42,43,44,45,46,47,56,57,58,59,60,61,62,63,64,65,66,67,68,69]
Eye	physiological and behavioral	24	[26,46,47,48,60,61,62,63,64,65,66,70,71,72,73,74,75,76,77,78,79,80,81,82]
Electroencephalogram	physiological	20	[35,36,37,38,39,40,54,55,56,83,84,85,86,87,88,89,90,91,92,93]
Head	behavioral	17	[42,46,59,60,61,62,63,64,65,66,77,78,79,94,95,96,97]
Respiration	physiological	9	[27,29,33,36,42,47,57,58,60]
Assessment test	indirectly derived human-signals	8	[26,30,31,44,47,65,80,98]
Skin temperature	physiological	7	[33,37,47,57,61,62,65]
Electrogastrogram	physiological	6	[28,45,53,60,65,99]
Center of pressure	physiological	6	[35,65,98,100,101,102]
Electromyogram	physiological	5	[35,47,55,57,80]
Functional near- infrared spectroscopy	physiological	3	[103,104,105]
Functional magnetic resonance imaging	physiological	2	[52,53]
Blood pressure	physiological	2	[53,57]
Impedance cardiography	physiological	1	[57]
Gait	behavioral	1	[106]
Body	behavioral	1	[59]
Endocrine	physiological	1	[53]

^1^ ECG and PPG are combined into a single sensor domain as they share major features.

**Table 2:** Selected multimodal human-signal sensors and measurement specifications.

Sensor Domain	Sensor	Measurement ^1^	Sampling Rate (Hz)
PPG	Shimmer3 GSR+	Voltage (mV)	128
EDA	Shimmer3 GSR+	Skin resistance (kΩ)	128
Eye	HoloLens 2 ^2^	Eye position and gaze direction	30
EEG	Enobio 8	Voltage (μV), used channels ^3^: F3, F4, C3, C4, P3, P4, O1, O2	500
SKT	Core body temperature	Temperature (°C)	1
Head	HoloLens 2	Head position and rotation	30
Head	IMU (WT901BLECL)	Head acceleration and angular velocity	4
Body	Azure Kinect	Tracked body joints	30

^1^ The acquisition system was additionally designed to collect facial expression (iPad), hand tracking (HoloLens 2), viewport image (HoloLens 2–WebRTC), and simulator motion (inertial measurement unit, IMU), although these signals were not analyzed in this study. ^2^ Eye data was collected from the left, right, and center using an extended eye tracking library. ^3^ Electrode placement followed the International 10–20 system.

**Table 3:** Design of driving scenarios for inducing CMS.

Step	Scenario	Duration	Description
Start	straight acceleration	2 min	0 → 40 km/h
1	straight constant velocity	4 min	40 km/h
2	straight acceleration or deceleration	4 min	(1) [2 times] 40 → −40 km/h (30 s), −40 → 40 km/h (30 s)
			(2) 40 → 20 km/h (10 s)
			(3) [2 times] 20 → 60 km/h (20 s), 60 → 20 km/h (20 s)
			(4) 20 → 60 km/h (20 s)
			(5) 60 → 40 km/h (10 s)
3	constant velocity curve (velocity = 40 km/h)	4 min	(1) [2 times] min radius = 135 m (≈total 90°), left curve (30 s) → right curve (30 s)
			(2) [6 times] min radius = 60 m (≈total 67.5°), left curve (10 s) → right curve (10 s)
4	constant velocity slope	4 min	(1) [2 times] slope = 9% (≈max pitch 5.1°), up slope (30 s) → down slope (30 s)
			(2) [6 times] slope = 9% (≈max pitch 5.1°), up slope (10 s) → down slope (10 s)
End	straight deceleration	2 min	40 → 0 km/h

**Table 4:** Extracted features by sensor modality: signal-wise (SwF) and window-derived (WdF).

Sensor Modality	SwF ^1^	WdF ^1^
PPG	whole, LF, and HF band signals	HR, IBI, LF/HF, HRV, SDNN, RMSSD, SD1, SD2, SD1/SD2, CSI, CVI, CSI/CVI, BR
EDA	whole, SCL, SCR, and Posada-Quintero LF ^2^ band signals	–
EEG	whole and standard frequency band signals (δ, θ, α, β, γ) by brain region (global, frontal, central, parietal, occipital)	β/α, (α + θ)/β, θ/α, θ/β, (α + θ)/(α + β), Fθ/Pα, FBN metrics (coherence, PLV ^3^; 8 ch × 8 ch adjacency = 28 edges) computed from whole and standard frequency band signals
SKT	whole band signal	–
Eye ^4^	eye closure signals (L + R, L × R), convergence distance, gaze direction (yaw, pitch), gaze velocity (°/s)	blink rate, vergence loss ratio, saccade ratio, fixation ratio, VOR, path length, heatmap entropy (visual entropy)
Head (HoloLens 2)	positions (sway, heave, surge), rotations (pitch, yaw, roll), direction (yaw, pitch)	–
Head (IMU)	accelerations (sway, heave, roll), angular velocities (pitch, yaw, roll)	–

^1^ Most abbreviations used in this table are defined in Section S1 (Supplementary Materials). Abbreviations not defined in Section S1 are expanded at their first appearance in this table. ^2^ Posada-Quintero et al. [138]. ^3^ Phase locking value. ^4^ L = left, R = right.

**Table 5:** MSSQ-Short percentile scores by susceptibility tertile and gender.

Gender	Low (0–33%)	Moderate (33–66%)	High (66–100%)
Total	(*N* = 13) Mean = 18.41, SD = 11.58	(*N* = 26) Mean = 52.54, SD = 7.89	(*N* = 51) Mean = 85.18, SD = 10.24
Male	(*N* = 11) Mean = 19.11, SD = 10.73	(*N* = 19) Mean = 51.15, SD = 7.67	(*N* = 34) Mean = 84.81, SD = 10.09
Female	(*N* = 2) Mean = 14.52, SD = 20.54	(*N* = 7) Mean = 56.32, SD = 7.75	(*N* = 17) Mean = 85.91, SD = 10.82

*N* = number of participants.

**Table 6:** Mean and standard deviation of ΔSSQ for the different MS types.

S-VIMS Group	Mean	SD	F-VIMS Group	Mean	SD
CMS	T: 16.065	T: 16.853	CMS	T: 19.107	T: 20.968
	N: 7.805	N: 13.377		N: 11.407	N: 18.933
	O: 17.916	O: 16.025		O: 20.763	O: 19.004
	D: 15.502	D: 23.869		D: 16.341	D: 26.461
VIMS	T: 12.155	T: 16.552	VIMS	T: 8.130	T: 15.893
	N: 6.288	N: 9.855		N: 5.600	N: 12.652
	O: 13.437	O: 18.843		O: 7.086	O: 16.942
	D: 11.389	D: 21.496		D: 9.078	D: 21.806
Co-MS	T: 22.185	T: 27.464	Co-MS	T: 21.139	T: 17.983
	N: 13.876	N: 26.624		N: 15.347	N: 17.817
	O: 23.429	O: 25.157		O: 18.950	O: 16.433
	D: 22.185	D: 29.600		D: 21.485	D: 26.849

T = total, N = nausea, O = oculomotor, D = disorientation.

**Table 7:** Three-way mixed RM ANOVA results for the effects of the MS type, gender, and susceptibility on ΔSSQ in the S-VIMS Group.

Effect	ΔSSQ_T_			ΔSSQ_N_			ΔSSQ_O_			ΔSSQ_D_		
	*F*	*η* ^2^ *_G_*	*p*	*F*	*η* ^2^ *_G_*	*p*	*F*	*η* ^2^ *_G_*	*p*	*F*	*η* ^2^ *_G_*	*p*
Gender	0.404	0.004	0.711	1.296	0.012	0.826	0.033	<0.001	0.856	0.283	0.003	0.874
Susceptibility	5.345	0.054	0.182	1.995	0.019	0.826	5.781	0.064	0.146	3.890	0.039	0.389
Gender × Susceptibility	0.274	0.003	0.711	0.027	<0.001	0.871	0.558	0.007	0.666	0.379	0.004	0.874
MS type	1.287	0.018	0.711	0.706	0.011	0.870	1.584	0.020	0.531	0.715	0.010	0.874
MS type × Gender	0.308	0.004	0.736	1.052	0.016	0.826	0.165	0.002	0.856	0.093	0.001	0.912
MS type × Susceptibility	0.608	0.009	0.711	0.385	0.006	0.871	0.751	0.010	0.666	0.290	0.004	0.874
MS type × Gender × Susceptibility	0.499	0.007	0.711	0.243	0.004	0.871	1.509	0.019	0.531	0.397	0.006	0.874

T = total, N = nausea, O = oculomotor, D = disorientation. Effect size is reported as *η*^2^*_G_*. *p*-values were adjusted using the Benjamini–Hochberg procedure.

**Table 8:** Three-way mixed RM ANOVA results for the effects of the MS type, gender, and susceptibility on ΔSSQ in the F-VIMS Group.

Effect	ΔSSQ_T_			ΔSSQ_N_			ΔSSQ_O_			ΔSSQ_D_		
	*F*	*η* ^2^ *_G_*	*p*	*F*	*η* ^2^ *_G_*	*p*	*F*	*η* ^2^ *_G_*	*p*	*F*	*η* ^2^ *_G_*	*p*
Gender	5.078	0.060	† 0.069	9.804	0.090	* 0.011	3.314	0.039	0.177	1.126	0.015	0.412
Susceptibility	0.218	0.003	0.750	2.332	0.023	0.235	0.031	<0.001	0.862	0.063	<0.001	0.804
Gender × Susceptibility	3.109	0.038	0.149	6.510	0.062	* 0.034	0.750	0.009	0.685	2.046	0.027	0.300
MS type	9.923	0.100	* 0.001	6.166	0.078	* 0.011	9.263	0.097	* 0.002	5.748	0.056	* 0.028
MS type × Gender	4.611	0.049	* 0.044	1.619	0.022	0.286	3.463	0.039	0.126	5.102	0.050	* 0.028
MS type × Susceptibility	0.184	0.002	0.832	0.187	0.003	0.829	0.555	0.006	0.806	0.842	0.009	0.507
MS type × Gender × Susceptibility	0.411	0.010	0.576	1.086	0.015	0.399	0.156	0.002	0.862	1.801	0.018	0.300

T = total, N = nausea, O = oculomotor, D = disorientation. Effect size is reported as *η*^2^*_G_*. *p*-values were adjusted using Benjamini–Hochberg procedure (*: *p* < 0.05, †: *p* < 0.1).

**Table 9:** Three-way ANOVA results for the effects of single-stimulus VIMS type, gender, and susceptibility on ΔSSQ.

Effect	ΔSSQ_T_			ΔSSQ_N_			ΔSSQ_O_			ΔSSQ_D_		
	*F*	*η* ^2^ *_G_*	*p*	*F*	*η* ^2^ *_G_*	*p*	*F*	*η* ^2^ *_G_*	*p*	*F*	*η* ^2^ *_G_*	*p*
VIMS type	0.147	0.002	0.703	0.232	0.003	0.632	1.118	0.013	0.763	0.034	<0.001	0.855
Gender	1.917	0.023	0.654	2.975	0.035	0.357	1.223	0.015	0.763	0.733	0.009	0.680
Susceptibility	0.534	0.006	0.654	0.293	0.004	0.632	0.912	0.011	0.763	0.979	0.012	0.680
VIMS type × Gender	1.242	0.015	0.654	1.280	0.015	0.457	0.504	0.006	0.763	1.346	0.016	0.680
VIMS type × Susceptibility	0.533	0.006	0.654	2.081	0.025	0.357	0.148	0.002	0.766	0.121	0.001	0.850
Gender × Susceptibility	0.752	0.009	0.654	0.403	0.005	0.632	0.370	0.004	0.763	1.008	0.012	0.680
VIMS type × Gender × Susceptibility	0.241	0.003	0.703	2.183	0.026	0.357	0.089	0.001	0.766	0.490	0.006	0.680

T = total, N = nausea, O = oculomotor, D = disorientation. Effect size is reported as *η*^2^*_G_*. *p*-values were adjusted using the Benjamini–Hochberg procedure.

**Table 10:** Three-way ANOVA results for the effects of the combined-stimulus VIMS type, gender, and susceptibility on ΔSSQ in the Co-MS condition.

Effect	ΔSSQ_T_			ΔSSQ_N_			ΔSSQ_O_			ΔSSQ_D_		
	*F*	*η* ^2^ *_G_*	*p*	*F*	*η* ^2^ *_G_*	*p*	*F*	*η* ^2^ *_G_*	*p*	*F*	*η* ^2^ *_G_*	*p*
VIMS type	1.568	0.019	0.266	3.022	0.036	0.182	0.090	0.001	0.872	1.746	0.021	0.270
Gender	1.742	0.021	0.266	0.484	0.006	0.570	1.760	0.021	0.660	1.723	0.021	0.270
Susceptibility	1.286	0.015	0.266	0.053	0.001	0.818	2.838	0.033	0.232	0.647	0.008	0.424
VIMS type × Gender	6.313	0.071	† 0.098	7.692	0.086	* 0.048	4.021	0.047	0.232	2.420	0.029	0.270
VIMS type × Susceptibility	3.740	0.044	0.198	2.701	0.032	0.182	2.780	0.033	0.232	2.524	0.030	0.270
Gender × Susceptibility	1.253	0.015	0.266	3.140	0.037	0.182	0.026	<0.001	0.872	1.158	0.014	0.332
VIMS type × Gender × Susceptibility	1.777	0.021	0.266	1.136	0.014	0.406	0.724	0.009	0.556	2.489	0.029	0.270

T = total, N = nausea, O = oculomotor, D = disorientation. Effect size is reported as *η*^2^*_G_*. *p*-values were adjusted using Benjamini–Hochberg procedure (*: *p* < 0.05, †: *p* < 0.1).

**Table 11:** Mean and standard deviation of IEQ scores by VIMS type.

S-VIMS Group	Mean	SD	F-VIMS Group	Mean	SD
VIMS	Cap: 39.523	Cap: 4.810	VIMS	Cap: 38.659	Cap: 6.911
	Dis: 7.523	Dis: 2.246		Dis: 7.705	Dis: 2.041
	Com: 10.977	Com: 2.881		Com: 0.795	Com: 3.062
	Tra: 14.682	Tra: 3.010		Tra: 14.409	Tra: 3.090
Co-MS	Cap: 39.543	Cap: 7.384	Co-MS	Cap: 38.978	Cap: 8.068
	Dis: 6.761	Dis: 2.068		Dis: 6.804	Dis: 2.400
	Com: 11.304	Com: 3.926		Com: 11.196	Com: 3.816
	Tra: 14.978	Tra: 3.201		Tra: 15.522	Tra: 3.160

Cap = captivation, Dis = real-world dissociation, Com = comprehension, Tra = transportation.

**Table 12:** Two-way mixed ANOVA results for the effects of the MS type and VIMS type on the IEQ scores.

Effect	IEQ_Cap_			IEQ_Dis_			IEQ_Com_			IEQ_Tra_		
	*F*	*η* ^2^ *_G_*	*p*	*F*	*η* ^2^ *_G_*	*p*	*F*	*η* ^2^ *_G_*	*p*	*F*	*η* ^2^ *_G_*	*p*
MS type	1.219	0.003	0.897	0.254	<0.001	0.132	0.186	<0.001	0.913	0.219	<0.001	0.342
VIMS type	0.017	<0.001	0.818	4.170	0.035	0.754	0.314	0.003	0.913	1.473	0.013	0.641
MS type × VIMS type	0.054	<0.001	0.897	0.099	<0.001	0.754	0.012	<0.001	0.913	1.748	0.004	0.342

Cap = captivation, Dis = real-world dissociation, Com = comprehension, Tra = transportation. Effect size is reported as *η*^2^*_G_*. *p*-values were adjusted using the Benjamini–Hochberg procedure.

**Table 13:** EEG features with significant correlations to the whole and standard band signals (*p* < 0.05 and |*r*| > 0.20). Bold indicates |*r*| > 0.25.

Features			MSL	*r*	*p*	Features			MSL	*r*	*p*
whole	global	ent	dO	−0.226	0.004	θ	frontal	ent	dT	−0.201	0.010
whole	global	skw	dD	0.212	0.007	θ	frontal	ent	dO	−0.258	<0.001
whole	global	psd_ent	dO	−0.241	0.002	θ	frontal	kut	dO	0.226	0.004
**whole**	**frontal**	**ent**	**dO**	**−0.250**	**0.001**	θ	frontal	psd_ent	dO	−0.204	0.009
whole	frontal	psd_ent	dO	−0.210	0.007	θ	central	ent	dT	−0.212	0.007
**whole**	**central**	**ent**	**dO**	**−0.255**	**0.001**	θ	central	ent	dO	−0.248	0.001
whole	central	psd_ent	dO	−0.216	0.006	θ	central	kut	dO	0.230	0.003
whole	parietal	ent	dO	−0.210	0.007	θ	central	psd_ent	dO	−0.210	0.007
whole	parietal	psd_ent	dO	−0.204	0.009	θ	parietal	ent	dO	−0.221	0.005
**whole**	**occipital**	**ent**	**dO**	**−0.258**	**<0.001**	θ	parietal	kut	dO	0.209	0.008
whole	occipital	kut	dO	0.205	0.009	θ	occipital	ent	dO	−0.241	0.002
whole	occipital	skw	dO	0.218	0.005	**θ**	**occipital**	**kut**	**dO**	**0.260**	**<0.001**
**whole**	**occipital**	**psd_ent**	**dO**	**−0.256**	**<0.001**	θ	occipital	skw	dT	0.216	0.006
δ	frontal	ent	dO	−0.234	0.003	θ	occipital	skw	dO	0.244	0.002
δ	frontal	kut	dT	0.206	0.009	α	frontal	ent	dT	−0.218	0.005
δ	frontal	kut	dO	0.245	0.002	**α**	**frontal**	**ent**	**dO**	**−0.263**	**<0.001**
δ	central	ent	dT	−0.225	0.004	α	central	ent	dT	−0.224	0.004
**δ**	**central**	**ent**	**dO**	**−0.257**	**<0.001**	**α**	**central**	**ent**	**dO**	**−0.252**	**0.001**
δ	central	kut	dT	0.207	0.008	α	parietal	ent	dO	−0.203	0.009
**δ**	**central**	**kut**	**dO**	**0.250**	**0.001**	α	occipital	ent	dT	−0.209	0.008
δ	central	psd_ent	dO	−0.200	0.011	α	occipital	ent	dO	−0.245	0.002
δ	parietal	ent	dT	−0.219	0.005	α	occipital	psd_ent	dO	−0.210	0.007
δ	parietal	ent	dN	−0.204	0.009	γ	global	mean	dD	0.237	0.002
δ	parietal	ent	dO	−0.231	0.003	WdF	β/(α + θ)	dT	−0.211	0.007	
δ	parietal	kut	dO	0.217	0.005	WdF	β/(α + θ)	dO	−0.243	0.002	
δ	occipital	ent	dT	−0.202	0.010	WdF	(α + θ)/(α + β)	dO	0.217	0.006	
δ	occipital	ent	dO	−0.246	0.002	WdF	(δ) Coh	F3-F4	dN	0.205	0.009
δ	occipital	kut	dO	0.223	0.004	WdF	(δ) PLV	O1-O2	dD	−0.205	0.009
δ	occipital	skw	dO	0.227	0.004	WdF	(θ) PLV	O1-O2	dD	−0.201	0.010
θ	global	mean	dN	0.242	0.002						

psd_ent = PSD entropy, ent = sample entropy, kut = kurtosis, skw = skewness, ptp = peak-to-peak amplitude. dT = ΔSSQ_T_, dN = ΔSSQ_N_, dO = ΔSSQ_O_, dD = ΔSSQ_D_.

**Table 14:** PPG features with significant correlations to the whole band and sub-band signals (medium effect size; *p* < 0.05 and |*r*| > 0.20).

Features		MSL	*r*	*p*
whole	var	dT	0.210	0.006
whole	var	dN	0.226	0.003
whole	var	dO	0.215	0.005
LF	var	dN	0.214	0.005
HF	var	dT	0.206	0.007
HF	var	dN	0.230	0.003
HF	var	dO	0.203	0.008
WdF	SD1/SD2	dT	−0.237	0.002
WdF	SD1/SD2	dO	−0.235	0.002
WdF	SD1/SD2	dD	−0.210	0.006
WdF	BR	dN	−0.201	0.009

var = variance. dT = ΔSSQ_T_, dN = ΔSSQ_N_, dO = ΔSSQ_O_, dD = ΔSSQ_D_.

**Table 15:** EDA features with significant correlations to the whole band and sub-band signals (*p* < 0.05 and |*r*| > 0.25). Bold indicates |*r*| > 0.30.

Features		MSL	*r*	*p*	Features		MSL	*r*	*p*
whole	var	dT	−0.289	<0.001	SCL	band_power	dD	−0.263	<0.001
**whole**	**var**	**dN**	**−0.321**	**<0.001**	SCR	var	dT	−0.291	<0.001
whole	var	dD	−0.268	<0.001	**SCR**	**var**	**dN**	**−0.323**	**<0.001**
whole	skw	dT	−0.251	0.001	SCR	ptp	dT	−0.285	<0.001
whole	skw	dN	−0.274	<0.001	**SCR**	**ptp**	**dN**	**−0.319**	**<0.001**
whole	ptp	dT	−0.279	<0.001	SCR	ptp	dD	−0.254	<0.001
**whole**	**ptp**	**dN**	**−0.309**	**<0.001**	SCR	band_power	dT	−0.275	<0.001
whole	ptp	dD	−0.267	<0.001	**SCR**	**band_power**	**dN**	**−0.306**	**<0.001**
whole	band_power	dT	−0.277	<0.001	SCR	band_power	dD	−0.260	<0.001
whole	band_power	dN	−0.309	<0.001	LF	var	dT	−0.295	<0.001
whole	band_power	dD	−0.260	<0.001	**LF**	**var**	**dN**	**−0.327**	**<0.001**
SCL	var	dT	−0.293	<0.001	LF	var	dD	−0.264	<0.001
**SCL**	**var**	**dN**	**−0.325**	**<0.001**	LF	ptp	dT	−0.284	<0.001
SCL	var	dD	−0.266	<0.001	**LF**	**ptp**	**dN**	**−0.318**	**<0.001**
SCL	ptp	dT	−0.282	<0.001	LF	ptp	dD	−0.258	<0.001
**SCL**	**ptp**	**dN**	**−0.317**	**<0.001**	LF	band_power	dT	−0.270	<0.001
SCL	ptp	dD	−0.254	<0.001	**LF**	**band_power**	**dN**	**−0.300**	**<0.001**
SCL	band_power	dT	−0.272	<0.001	LF	band_power	dD	−0.260	<0.001
**SCL**	**band_power**	**dN**	**−0.302**	**<0.001**	LF	band_power	dD	−0.260	<0.001

var = variance, skw = skewness, ptp = peak-to-peak amplitude. dT = ΔSSQ_T_, dN = ΔSSQ_N_, dO = ΔSSQ_O_, dD = ΔSSQ_D_.

**Table 16:** Eye features with significant correlations (medium effect size; *p* < 0.05 and |*r*| > 0.20). Bold indicates |*r*| > 0.25).

Features		MSL	*r*	*p*
Convergence distance	kut	dO	−0.201	0.009
Dir_yaw	mean	dO	−0.212	0.006
**Dir_yaw**	**psd_ent**	**dT**	**0.304**	**0.002**
Dir_yaw	psd_ent	dN	0.209	0.032
**Dir_yaw**	**psd_ent**	**dO**	**0.350**	**<0.001**
Dir_pitch	psd_ent	dT	0.210	0.032
**Dir_pitch**	**psd_ent**	**dO**	**0.283**	**0.003**
Velocity	mean	dT	0.206	0.007
Velocity	mean	dO	0.241	0.002
Velocity	skw	dO	−0.208	0.033
Velocity	psd_ent	dO	−0.206	0.035
WdF	accumulated degree	dT	0.205	0.007
WdF	accumulated degree	dO	0.241	0.002

Dir = direction, psd_ent = Shannon entropy of PSD, var = variance, kut = kurtosis, skew = skewness. dT = ΔSSQ_T_, dN = ΔSSQ_N_, dO = ΔSSQ_O_, dD = ΔSSQ_D_.

**Table 17:** Head features with significant correlations (large; *p* < 0.05 and |*r*| > 0.3). Bold indicates |r| > 0.35.

Features		MSL	*r*	*p*	Features		MSL	*r*	*p*
Pos_sway	var	dO	0.319	<0.001	Rot_pitch	ptp	dO	0.370	<0.001
Pos_sway	ptp	dT	0.314	<0.001	Rot_pitch	band_power	dT	0.346	<0.001
Pos_sway	ptp	dO	0.336	<0.001	Rot_pitch	band_power	dO	0.331	<0.001
Pos_sway	band_power	dO	0.319	<0.001	Dir_pitch	var	dT	0.347	<0.001
Pos_heav	var	dO	0.306	<0.001	Dir_pitch	var	dO	0.329	<0.001
Pos_heav	ptp	dT	0.319	<0.001	**Dir_pitch**	**ptp**	**dT**	**0.363**	**<0.001**
Pos_heav	ptp	dO	0.337	<0.001	**Dir_pitch**	**ptp**	**dO**	**0.369**	**<0.001**
Pos_heav	band_power	dO	0.301	<0.001	Dir_pitch	band_power	dT	0.345	<0.001
Pos_surg	ent	dT	−0.318	<0.001	Dir_pitch	band_power	dO	0.332	<0.001
Pos_surg	ent	dO	−0.325	<0.001	Acc_heave	ent	dT	−0.345	<0.001
Pos_surg	var	dT	0.331	<0.001	Acc_heave	ent	dO	−0.347	<0.001
**Pos_surg**	**var**	**dO**	**0.380**	**<0.001**	Acc_surge	ptp	dT	0.345	<0.001
**Pos_surg**	**ptp**	**dT**	**0.352**	**<0.001**	**Acc_surge**	**ptp**	**dO**	**0.369**	**<0.001**
**Pos_surg**	**ptp**	**dO**	**0.384**	**<0.001**	AngV_pitch	var	dO	0.306	<0.001
**Pos_surg**	**band_power**	**dT**	**0.353**	**<0.001**	AngV_pitch	ptp	dT	0.317	<0.001
**Pos_surg**	**band_power**	**dO**	**0.394**	**<0.001**	AngV_pitch	ptp	dO	0.332	<0.001
**Pos_surg**	**psd_ent**	**dT**	**−0.359**	**<0.001**	AngV_yaw	kut	dT	−0.307	<0.001
**Pos_surg**	**psd_ent**	**dO**	**−0.363**	**<0.001**	AngV_yaw	kut	dO	−0.334	<0.001
Rot_pitch	var	dT	0.347	<0.001	AngV_roll	kut	dT	−0.306	<0.001
Rot_pitch	var	dO	0.331	<0.001	AngV_roll	kut	dO	−0.327	<0.001
**Rot_pitch**	**ptp**	**dT**	**0.364**	**<0.001**					

Pos = position, Rot = rotation, Dir = direction, Acc = acceleration, AngV = angular velocity, psd_ent = PSD entropy, ent = Sample entropy, var = variance, ptp = peak-to-peak amplitude, kut = kurtosis. dT = ΔSSQ_T_, dN = ΔSSQ_N_, dO = ΔSSQ_O_, dD = ΔSSQ_D_.

**Table 18:** Significant correlations between all features and fast motion sickness scale (medium effect size; *p* < 0.05 and |*r*| > 0.20).

Features			r	*p*
Head	Pos_heav	ptp	0.247	<0.001
Head	Pos_surg	ptp	0.244	<0.001
Head	Pos_surg	psd_ent	−0.210	<0.001
Head	Rot_pitch	ptp	0.226	<0.001
Head	Rot_roll	ptp	0.206	<0.001
Head	Dir_pitch	ptp	0.224	<0.001
Head	Acc_sway	ptp	0.211	<0.001
Head	Acc_surge	ptp	0.206	<0.001
Head	AngV_pitch	ptp	0.214	<0.001

Pos = position, Rot = rotation, Acc = acceleration, AngV = angular velocity, heav = heave, surg = surge, psd_ent = PSD entropy, ptp = peak-to-peak amplitude.

## Data Availability

The preprocessing and feature-extraction procedures are fully specified in the manuscript. The feature dataset and the analysis code are publicly available in the GitHub repository at https://github.com/sgkim6326/2025-NRF-Multimodal-MS-Analysis (accessed on 19 February 2026). These datasets are licensed under the Creative Commons Attribution 4.0 International License (CC BY 4.0). The raw multimodal physiological recordings are not publicly available because they contain personal health-related information and are extremely large; however, the full dataset is available on request from the corresponding author.

## Supplementary Materials

The following supporting information can be downloaded at: https://www.mdpi.com/article/10.3390/s26051675/s1, Section S1: Detailed human-signal features by sensor domain in the HS-Set; Section S2: Technical details of the autonomous driving simulator; Section S3: Data synchronization and system I/O optimization; Figure S1: Motion profiles and vehicle trajectories for different driving scenarios; Figure S2: Process of road and surrounding environment generation based on vehicle trajectories; Figure S3: Operator console interface for real-time monitoring and remote recording; Table S1: Specific search queries and results for each database; Table S2: Summary of ECG and PPG features (not mutually exclusive); Table S3: Summary of EDA features (not mutually exclusive); Table S4: Summary of Eye features (not mutually exclusive); Table S5: Summary of EEG features (not mutually exclusive); Table S6: Summary of Head features (not mutually exclusive); Table S7: Summary of EGG features (not mutually exclusive); Table S8: Summary of miscellaneous features (not mutually exclusive); Table S9: Cutoff frequencies and scale factors used in the MCA; Table S10: Lower and upper cutoff frequencies for EEG preprocessing band-pass filters in the HS-Set; Table S11: Lower and upper cutoff frequencies for the EEG decomposition bands (δ-γ) in the HS-Set.

## References

[1] SAE International (2021). Taxonomy and Definitions for Terms Related to Driving Automation Systems for On-Road Motor Vehicles.

[2] Ataya A., Kim W., Elsharkawy A., Kim S. (2021). How to interact with a fully autonomous vehicle: Naturalistic ways for drivers to intervene in the vehicle system while performing non-driving related tasks. Sensors.

[3] Schartmüller C., Weigl K., Löcken A., Wintersberger P., Steinhauser M., Riener A. (2021). Displays for productive non-driving related tasks: Visual behavior and its impact in conditionally automated driving. Multimodal Technol. Interact..

[4] Yoon S.H., Ji Y.G. (2019). Non-driving-related tasks, workload, and takeover performance in highly automated driving contexts. Transp. Res. Part F Traffic Psychol. Behav..

[5] Müller A.L., Fernandes-Estrela N., Hetfleisch R., Zecha L., Abendroth B. (2021). Effects of non-driving related tasks on mental workload and take-over times during conditional automated driving. Eur. Transp. Res. Rev..

[6] Suwa T., Sato Y., Wada T. (2022). Reducing motion sickness when reading with head-mounted displays by using see-through background images. Front. Virtual Real..

[7] Pfleging B., Rang M., Broy N. Investigating user needs for non-driving-related activities during automated driving. Proceedings of the International Conference on Mobile and Ubiquitous Multimedia (MUM).

[8] Detjen H., Pfleging B., Schneegass S. A wizard of oz field study to understand non-driving-related activities, trust, and acceptance of automated vehicles. Proceedings of the International Conference on Automotive User Interfaces and Interactive Vehicular Applications (AutomotiveUI).

[9] Reed M.P., Ebert S.M., Jones M.L. (2020). Naturalistic Passenger Behavior: Posture and Activities.

[10] Li J., George C., Ngao A., Holländer K., Mayer S., Butz A. An exploration of users’ thoughts on rear-seat productivity in virtual reality. Proceedings of the International Conference on Automotive User Interfaces and Interactive Vehicular Applications (AutomotiveUI).

[11] Elsharkawy A.I.A.M., Ataya A.A.S., Yeo D., An E., Hwang S., Kim S. SYNC-VR: Synchronizing your senses to conquer motion sickness for enriching in-vehicle virtual reality. Proceedings of the CHI Conference on Human Factors in Computing Systems.

[12] Diels C., Bos J.E. (2016). Self-driving carsickness. Appl. Ergon..

[13] Metzulat M., Metz B., Landau A., Neukum A., Kunde W. (2024). Does the visual input matter? Influence of non-driving related tasks on car sickness in an open road setting. Transp. Res. Part F Traffic Psychol. Behav..

[14] Turner M. (1999). Motion sickness in public road transport: Passenger behaviour and susceptibility. Ergonomics.

[15] Xie W., He D., Wu G. (2023). Inducers of motion sickness in vehicles: A systematic review of experimental evidence and meta-analysis. Transp. Res. Part F Traffic Psychol. Behav..

[16] Kennedy R.S., Lane N.E., Berbaum K.S., Lilienthal M.G. (1993). Simulator sickness questionnaire: An enhanced method for quantifying simulator sickness. Int. J. Aviat. Psychol..

[17] Keshavarz B., Hecht H. (2011). Validating an efficient method to quantify motion sickness. Hum. Factors.

[18] Bos J.E., MacKinnon S.N., Patterson A. (2005). Motion sickness symptoms in a ship motion simulator: Effects of inside, outside, and no view. Aviat. Space Environ. Med..

[19] (1997). Mechanical Vibration and Shock—Evaluation of Human Exposure to Whole-Body Vibration—Part 1: General Requirements.

[20] Asua E., Gutiérrez-Zaballa J., Mata-Carballeira O., Ruiz J.A., del Campo I. (2022). Analysis of the motion sickness and the lack of comfort in car passengers. Appl. Sci..

[21] Htike Z., Papaioannou G., Siampis E., Velenis E., Longo S. (2021). Fundamentals of motion planning for mitigating motion sickness in automated vehicles. IEEE Trans. Veh. Technol..

[22] Li D., Hu J. (2021). Mitigating motion sickness in automated vehicles with frequency-shaping approach to motion planning. IEEE Robot. Autom. Lett..

[23] Seo J.W., Ko C.H., Sung J.H., Yun D.G., Lee B., Kim J.S., Park T., Park H.S., Ju S.P., Chung C.C. Data-driven human modeling based on temporal information and nonlinear model predictive control for adaptive cruise control reducing motion sickeness. Proceedings of the IEEE International Conference on Intelligent Transportation Systems (ITSC).

[24] Golding J.F. (1998). Motion sickness susceptibility questionnaire revised and its relationship to other forms of sickness. Brain Res. Bull..

[25] Rigby J.M., Brumby D.P., Gould S.J., Cox A.L. Development of a questionnaire to measure immersion in video media: The Film IEQ. Proceedings of the ACM International Conference on Interactive Experiences for TV and Online Video (TVX).

[26] Ramaioli C., Steinmetzer T., Brietzke A., Meyer P., Pham Xuan R., Schneider E., Gorges M. (2023). Assessment of vestibulo-ocular reflex and its adaptation during stop-and-go car rides in motion sickness susceptible passengers. Exp. Brain Res..

[27] Paredes P.E., Balters S., Qian K., Murnane E.L., Ordóñez F., Ju W., Landay J.A. (2018). Driving with the fishes: Towards calming and mindful virtual reality experiences for the car. Proc. ACM Interact. Mob. Wearable Ubiquitous Technol..

[28] Schartmüller C., Riener A. Sick of scents: Investigating non-invasive olfactory motion sickness mitigation in automated driving. Proceedings of the International Conference on Automotive User Interfaces and Interactive Vehicular Applications (AutomotiveUI).

[29] Kojima T., Ohsuga M., Kamakura Y., Hori J., Watanabe S. Assessment of car sickness in passengers using physiological indices. Proceedings of the IEEE International Conference on Systems, Man, and Cybernetics (SMC).

[30] Aydin K., Kara E., Adatepe N.U., Atas A. (2024). Neuro-ophthalmic and neuro-otologic evaluation in individuals with motion sickness susceptibility. J. Int. Adv. Otol..

[31] Karababa E., Satar B., Genç H. (2023). Evaluation of effects of optokinetic and rotational stimuli with functional head impulse test (fHIT) in individuals with motion sickness. Eur. Arch. Oto-Rhino-Laryngol..

[32] Tamura A., Iwamoto T., Ozaki H., Kimura M., Tsujimoto Y., Wada Y. (2018). Wrist-worn electrodermal activity as a novel neurophysiological biomarker of autonomic symptoms in spatial disorientation. Front. Neurol..

[33] Rahimzadeh G., Lacy K., Mohamed S., Plawiak P., Sharifrazi D., Toomey N.G., Asadi H. Immediate detection of simulator sickness in virtual environments using integrated subjective feedback and physiological signals. Proceedings of the IEEE International Conference on E-health Networking, Application & Services (HealthCom).

[34] Kobayashi N., Yamazaki M., Mizutani R. Impact of visually induced motion sickness from VR depending on viewing patterns, view movement, and background motion. Proceedings of the Annual International Conference of the IEEE Engineering in Medicine and Biology Society (EMBC).

[35] Recenti M., Pescaglia F., Guerrini L., Maruotto I., Gelormini C., Jacob D., Aubonnet R., Petersen H., Gargiulo P. New frontiers in postural control and motion sickness assessment: The BioVRSea paradigm. Proceedings of the IEEE International Conference on Metrology for eXtended Reality, Artificial Intelligence and Neural Engineering (MetroXRAINE).

[36] Park S.-H., Han D.-K., Lee S.-W. Dynamic multi-modal fusion for biosignal-based motion sickness prediction in vehicles. Proceedings of the Annual International Conference of the IEEE Engineering in Medicine and Biology Society (EMBC).

[37] Su H., Jia Y. (2021). Study of human comfort in autonomous vehicles using wearable sensors. IEEE Trans. Intell. Transp. Syst..

[38] Lee S., Kim S., Kim H.G., Ro Y.M. (2021). Assessing individual VR sickness through deep feature fusion of VR video and physiological response. IEEE Trans. Circuits Syst. Video Technol..

[39] Sameri J., Coenegracht H., Van Damme S., De Turck F., Torres Vega M. (2024). Physiology-driven cybersickness detection in virtual reality: A machine learning and explainable AI approach. Virtual Real..

[40] Oh H., Son W. (2022). Cybersickness and its severity arising from virtual reality content: A comprehensive study. Sensors.

[41] Reddy A., Kim J.R. Assessment and quantification of virtual reality induced sickness in relation to age and gender: A multi-modal approach. Proceedings of the IEEE MIT Undergraduate Research Technology Conference (URTC).

[42] Kundu R.K., Islam R., Calyam P., Hoque K.A. TruVR: Trustworthy cybersickness detection using explainable machine learning. Proceedings of the IEEE International Symposium on Mixed and Augmented Reality (ISMAR).

[43] Martin N., Mathieu N., Pallamin N., Ragot M., Diverrez J.-M. Virtual reality sickness detection: An approach based on physiological signals and machine learning. Proceedings of the IEEE International Symposium on Mixed and Augmented Reality (ISMAR).

[44] Andre L., Coutellier R. Cybersickness and evaluation of a remediation system: A pilot study. Proceedings of the International Conference on 3D Immersion (IC3D).

[45] Gruden T., Popović N.B., Stojmenova K., Jakus G., Miljković N., Tomažič S., Sodnik J. (2021). Electrogastrography in autonomous vehicles—An objective method for assessment of motion sickness in simulated driving environments. Sensors.

[46] Islam R., Desai K., Quarles J. Towards forecasting the onset of cybersickness by fusing physiological, head-tracking and eye-tracking with multimodal deep fusion network. Proceedings of the IEEE International Symposium on Mixed and Augmented Reality (ISMAR).

[47] Kim H., Kim D.J., Chung W.H., Park K.-A., Kim J.D., Kim D., Kim K., Jeon H.J. (2021). Clinical predictors of cybersickness in virtual reality (VR) among highly stressed people. Sci. Rep..

[48] Hussain R., Chessa M., Solari F. (2021). Mitigating cybersickness in virtual reality systems through foveated depth-of-field blur. Sensors.

[49] Preciado C.E., Starrett M.J., Ekstrom A.D. (2021). Assessment of a short, focused training to reduce symptoms of cybersickness. Presence.

[50] Lee R., Kim Y.S. (2024). VR sickness evaluation method using recurrence period density entropy. Appl. Sci..

[51] Reyero Lobo P., Perez P. Heart rate variability for non-intrusive cybersickness detection. Proceedings of the ACM International Conference on Interactive Media Experiences (IMX).

[52] Ruffle J.K., Patel A., Giampietro V., Howard M.A., Sanger G.J., Andrews P.L., Williams S.C., Aziz Q., Farmer A.D. (2019). Functional brain networks and neuroanatomy underpinning nausea severity can predict nausea susceptibility using machine learning. J. Physiol..

[53] Farmer A.D., Ban V.F., Coen S.J., Sanger G.J., Barker G.J., Gresty M.A., Giampietro V.P., Williams S.C., Webb D.L., Hellström P.M. (2015). Visually induced nausea causes characteristic changes in cerebral, autonomic and endocrine function in humans. J. Physiol..

[54] Kim S., Lee S., Ro Y.M. Estimating VR sickness caused By camera shake in VR videography. Proceedings of the IEEE International Conference on Image Processing (ICIP).

[55] Recenti M., Ricciardi C., Aubonnet R., Picone I., Jacob D., Svansson H.Á., Agnarsdóttir S., Karlsson G.H., Baeringsdóttir V., Petersen H. (2021). Toward predicting motion sickness using virtual reality and a moving platform assessing brain, muscles, and heart signals. Front. Bioeng. Biotechnol..

[56] Lee S., Kim S., Kim H.G., Kim M.S., Yun S., Jeong B., Ro Y.M. Physiological fusion net: Quantifying individual VR sickness with content stimulus and physiological response. Proceedings of the IEEE International Conference on Image Processing (ICIP).

[57] Cowings P.S., Toscano W.B., Reschke M.F., Tsehay A. (2018). Psychophysiological assessment and correction of spatial disorientation during simulated Orion spacecraft re-entry. Int. J. Psychophysiol..

[58] Gavgani A.M., Nesbitt K.V., Blackmore K.L., Nalivaiko E. (2017). Profiling subjective symptoms and autonomic changes associated with cybersickness. Auton. Neurosci..

[59] Nunes da Silva W., Porcino T.M., Castanho C.D., Jacobi R.P. Analysis of cybersickness through biosignals: An approach with symbolic machine learning. Proceedings of the Symposium on Virtual and Augmented Reality (SVR).

[60] Dennison M.S., Wisti A.Z., D’Zmura M. (2016). Use of physiological signals to predict cybersickness. Displays.

[61] Hadadi A., Chardonnet J.-R., Guillet C., Ovtcharova J. Machine Learning Application for Real-Time Simulator. Proceedings of the International Conference on Machine Learning Technologies (ICMLT).

[62] Jeong D., Han K. (2024). Precyse: Predicting cybersickness using transformer for multimodal time-series sensor data. Proc. ACM Interact. Mob. Wearable Ubiquitous Technol..

[63] Kundu R.K., Elsaid O.Y., Calyam P., Hoque K.A. VR-LENS: Super learning-based cybersickness detection and explainable AI-guided deployment in virtual reality. Proceedings of the International Conference on Intelligent User Interfaces (IUI).

[64] Jeong D., Paik S., Noh Y., Han K. (2023). MAC: Multimodal, attention-based cybersickness prediction modeling in virtual reality. Virtual Real..

[65] Li C.-C., Zhang Z.-R., Liu Y.-H., Zhang T., Zhang X.-T., Wang H., Wang X.-C. (2022). Multi-dimensional and objective assessment of motion sickness susceptibility based on machine learning. Front. Neurol..

[66] Jeong D., Han K. Leveraging multimodal sensory information in cybersickness prediction. Proceedings of the ACM Symposium on Virtual Reality Software and Technology (VRST).

[67] Arafat I.M., Ferdous S.M.S., Quarles J. The effects of cybersickness on persons with multiple sclerosis. Proceedings of the ACM Conference on Virtual Reality Software and Technology (VRST).

[68] Tang Q., Xiang H., Cheng J., Xiao X., Yu W., Zhang S., Tang B., Guo G. Study on evaluation method of motion sickness in electric vehicles. Proceedings of the CAA International Conference on Vehicular Control and Intelligence (CVCI).

[69] Deng Z., Yuan K., Xiao X. (2024). Investigative examination of motion sickness indicators for electric vehicles. Proc. Inst. Mech. Eng. Part D J. Automob. Eng..

[70] Shimada S., Pannattee P., Ikei Y., Nishiuchi N., Yem V. (2023). High-frequency cybersickness prediction using deep learning techniques with eye-related indices. IEEE Access.

[71] Lopes P., Tian N., Boulic R. Exploring blink-rate behaviors for cybersickness detection in VR. Proceedings of the IEEE Conference on Virtual Reality and 3D User Interfaces Abstracts and Workshops (VRW).

[72] Li Y., Pan L., Liu M., Shao Z., Xue M., Liao J., Zhao H., Wu M., Yu S., Wu X. (2024). Quantitative study on objective indicators for assessing motion sickness susceptibility based on Vestibulo-Ocular Reflex experiments. Sci. Rep..

[73] Josupeit J., Greim L. The optokinetic nystagmus as a physiological indicator of cybersickness–a vergence-based evaluation. Proceedings of the International Conference on Human-Computer Interaction.

[74] Fujikake K., Itadu Y., Takada H. Analyzing gaze data during rest time/driving simulator operation using machine learning. Proceedings of the International Conference on Human-Computer Interaction.

[75] Park S., Mun S., Ha J., Kim L. (2021). Non-contact measurement of motion sickness using pupillary rhythms from an infrared camera. Sensors.

[76] Lee J., Kim W., Kim J., Lee S. A study on virtual reality sickness and visual attention. Proceedings of the Asia-Pacific Signal and Information Processing Association Annual Summit and Conference (APSIPA ASC).

[77] Islam R., Desai K., Quarles J. Cybersickness prediction from integrated HMD’s sensors: A multimodal deep fusion approach using eye-tracking and head-tracking data. Proceedings of the IEEE International Symposium on Mixed and Augmented Reality (ISMAR).

[78] Tovar D., Wilmott J., Wu X., Martin D., Proulx M., Lindberg D., Zhao Y., Mercier O., Guan P. (2024). Identifying behavioral correlates to visual discomfort. ACM Trans. Graph..

[79] Fan C.-L., Hung T.-H., Hsu C.-H. (2022). Modeling the user experience of watching 360 videos with head-mounted displays. ACM Trans. Multimed. Comput. Commun. Appl..

[80] Berton B.R., Morel-Targosz C., Chaumillon R., Wolff M. Paving the way towards a methodology to faithfully assess physical, physiological, and cognitive impacts of augmented reality under constrained environments: A head-mounted display use case. Proceedings of the “Ergonomie et Informatique Avancée” Conference.

[81] Zhu H., Li T., Wang C., Jin W., Murali S., Xiao M., Ye D., Li M. (2022). Eyeqoe: A novel qoe assessment model for 360-degree videos using ocular behaviors. Proc. ACM Interact. Mob. Wearable Ubiquitous Technol..

[82] Lopes P., Tian N., Boulic R. Eye thought you were sick! exploring eye behaviors for cybersickness detection in VR. Proceedings of the ACM SIGGRAPH Conference on Motion, Interaction and Games (MIG).

[83] Hua C., Chai L., Yan Y., Liu J., Wang Q., Fu R., Zhou Z. (2023). Assessment of virtual reality motion sickness severity based on EEG via LSTM/BiLSTM. IEEE Sens. J..

[84] Liu M., Yang B., Xu M., Zan P., Chen L., Xia X. (2024). Exploring quantitative assessment of cybersickness in virtual reality using EEG signals and a CNN-ECA-LSTM network. Displays.

[85] Li Z., Zhao L., Chang J., Li W., Yang M., Li C., Wang R., Ji L. EEG-based evaluation of motion sickness and reducing sensory conflict in a simulated autonomous driving environment. Proceedings of the Annual International Conference of the IEEE Engineering in Medicine and Biology Society (EMBC).

[86] Qin B., Wu B., Zhou L., Chen Y., Qian Z., Zhu Q. Study on motion sickness based on EEG power spectrum characteristics. Proceedings of the IEEE International Conference on Medical Imaging Physics and Engineering (ICMIPE).

[87] Liu M., Yang B., Zan P., Chen L., Wang B., Xia X. (2024). Exploring the brain physiological activity and quantified assessment of VR cybersickness using EEG signals. Displays.

[88] Woo Y.S., Jang K.-M., Nam S.G., Kwon M., Lim H.K. (2023). Recovery time from VR sickness due to susceptibility: Objective and quantitative evaluation using electroencephalography. Heliyon.

[89] Hu H., Fang Z., Qian Z., Yao L., Tao L., Qin B. (2021). Stress assessment of vestibular endurance training for civil aviation flight students based on EEG. Front. Hum. Neurosci..

[90] Lim H.K., Ji K., Woo Y.S., Han D.-u., Lee D.-H., Nam S.G., Jang K.-M. (2021). Test-retest reliability of the virtual reality sickness evaluation using electroencephalography (EEG). Neurosci. Lett..

[91] Xu M., Yang B., Liu M., Huang Y., Xia X., Zhang J. SE-CNN attention structure for quantitative EEG-based assessment of VR motion sickness. Proceedings of the International Conference on Computing and Artificial Intelligence (ICCAI).

[92] Jang K.-M., Shin Woo Y., Kyoon Lim H. Electrophysiological changes in the virtual reality sickness: EEG in the VR sickness. Proceedings of the International Conference on 3D Web Technology (Web3D).

[93] Jeong D.K., Yoo S., Jang Y. VR sickness measurement with EEG using DNN algorithm. Proceedings of the ACM Symposium on Virtual Reality Software and Technology (VRST).

[94] Subudhi D., Balaji P., Muniyandi M. Objective quantification of circular vection in immersive environments. Proceedings of the International Conference on Human-Computer Interaction.

[95] Sugiura T., Wada T., Nagata T., Sakai K., Sato Y. (2019). Analysing effect of vehicle lean using cybernetic model of motion sickness. IFAC-PapersOnLine.

[96] Bala P., Oakley I., Nisi V., Nunes N.J. Dynamic field of view restriction in 360 video: Aligning optical flow and visual slam to mitigate VIMS. Proceedings of the CHI Conference on Human Factors in Computing Systems.

[97] Bala P., Oakley I., Nisi V., Nunes N. Staying on track: A comparative study on the use of optical flow in 360 video to mitigate VIMS. Proceedings of the ACM International Conference on Interactive Media Experiences (IMX).

[98] Laessoe U., Abrahamsen S., Zepernick S., Raunsbaek A., Stensen C. (2023). Motion sickness and cybersickness–Sensory mismatch. Physiol. Behav..

[99] Jakus G., Sodnik J., Miljković N. (2022). Electrogastrogram-derived features for automated sickness detection in driving simulator. Sensors.

[100] Xia Z., Liu Y., Bai Y., Zhang Y., Cheng C. (2024). Mismatches between 3D content acquisition and perception cause more visually induced motion sickness. Presence.

[101] Wang Y., Chardonnet J.-R., Merienne F. VR sickness prediction for navigation in immersive virtual environments using a deep long short term memory model. Proceedings of the IEEE Conference on Virtual Reality and 3D User Interfaces (VR).

[102] Choi C., Jun J., Heo J., Kim K. Effects of virtual-avatar motion-synchrony levels on full-body interaction. Proceedings of the ACM/SIGAPP Symposium on Applied Computing (SAC).

[103] Ren B., Guan W., Zhou Q. (2023). Study of motion sickness model based on fNIRS multiband features during car rides. Diagnostics.

[104] Pöhlmann K.M.T., Maior H.A., Föcker J., O’Hare L., Parke A., Ladowska A., Dickinson P. I think I don’t feel sick: Exploring the relationship between cognitive demand and cybersickness in virtual reality using fNIRS. Proceedings of the CHI Conference on Human Factors in Computing Systems.

[105] Yamamura H., Baldauf H., Kunze K. HemodynamicVR-adapting the user’s field of view during virtual reality locomotion tasks to reduce cybersickness using wearable functional near-infrared spectroscopy. Proceedings of the Augmented Humans International Conference (AHs).

[106] Feigl T., Roth D., Gradl S., Wirth M., Latoschik M.E., Eskofier B.M., Philippsen M., Mutschler C. (2019). Sick moves! motion parameters as indicators of simulator sickness. IEEE Trans. Vis. Comput. Graph..

[107] Cleij D., Venrooij J., Pretto P., Katliar M., Bülthoff H.H., Steffen D., Hoffmeyer F.W., Schöner H.-P. (2019). Comparison between filter-and optimization-based motion cueing algorithms for driving simulation. Transp. Res. Part F Traffic Psychol. Behav..

[108] Stahl K., Abdulsamad G., Leimbach K.-D., Vershinin Y.A. State of the art and simulation of motion cueing algorithms for a six degree of freedom driving simulator. Proceedings of the International IEEE Conference on Intelligent Transportation Systems (ITSC).

[109] Harm D.L., Reschke M.R., Parker D.E. (1999). Visual-Vestibular Integration Motion Perception Reporting.

[110] Kenward H., Pelligand L., Savary-Bataille K., Elliott J. (2015). Nausea: Current knowledge of mechanisms, measurement and clinical impact. Vet. J..

[111] Wickham R.J. (2020). Revisiting the physiology of nausea and vomiting—Challenging the paradigm. Support. Care Cancer.

[112] Irmak T., Kotian V., Happee R., de Winkel K.N., Pool D.M. (2022). Amplitude and temporal dynamics of motion sickness. Front. Syst. Neurosci..

[113] Kuiper O.X., Bos J.E., Diels C., Schmidt E.A. (2020). Knowing what’s coming: Anticipatory audio cues can mitigate motion sickness. Appl. Ergon..

[114] Lawther A., Griffin M.J. (1988). Motion sickness and motion characteristics of vessels at sea. Ergonomics.

[115] Palmisano S., Constable R. (2022). Reductions in sickness with repeated exposure to HMD-based virtual reality appear to be game-specific. Virtual Real..

[116] Risi D., Palmisano S. (2019). Effects of postural stability, active control, exposure duration and repeated exposures on HMD induced cybersickness. Displays.

[117] Stanney K.M., Hale K.S., Nahmens I., Kennedy R.S. (2003). What to expect from immersive virtual environment exposure: Influences of gender, body mass index, and past experience. Hum. Factors.

[118] Caserman P., Garcia-Agundez A., Gámez Zerban A., Göbel S. (2021). Cybersickness in current-generation virtual reality head-mounted displays: Systematic review and outlook. Virtual Real..

[119] Won J.-h., Na H.C., Kim Y.S. (2024). A new training method for VR sickness reduction. Appl. Sci..

[120] Dam A., Jeon M. A review of motion sickness in automated vehicles. Proceedings of the International Conference on Automotive User Interfaces and Interactive Vehicular Applications (AutomotiveUI).

[121] Henry E.H., Bougard C., Bourdin C., Bringoux L. (2023). Car sickness in real driving conditions: Effect of lateral acceleration and predictability reflected by physiological changes. Transp. Res. Part F Traffic Psychol. Behav..

[122] Lackner J.R., Graybiel A. (1986). The effective intensity of Coriolis, cross-coupling stimulation is gravitoinertial force dependent: Implications for space motion sickness. Aviat. Space Environ. Med..

[123] O’hanlon J.F., McCauley M.E. (1974). Motion sickness incidence as a function of the frequency and acceleration of vertical sinusoidal motion. Aerosp. Med..

[124] Wijlens R., van Paassen M.M., Mulder M., Takamatsu A., Makita M., Wada T. (2022). Reducing motion sickness by manipulating an autonomous vehicle’s accelerations. IFAC-PapersOnLine.

[125] Butler C.A., Griffin M.J. (2006). Motion sickness during fore-and-aft oscillation: Effect of the visual scene. Aviat. Space Environ. Med..

[126] Butler C., Griffin M.J. (2009). Motion sickness with combined fore-aft and pitch oscillation: Effect of phase and the visual scene. Aviat. Space Environ. Med..

[127] So R.H., Lo W., Ho A.T. (2001). Effects of navigation speed on motion sickness caused by an immersive virtual environment. Hum. Factors.

[128] Chang E., Seo D., Kim H.T., Yoo B. (2018). An integrated model of cybersickness: Understanding user’s discomfort in virtual reality. J. Korean Inst. Inf. Sci. Eng..

[129] Kim J., Oh H., Kim W., Choi S., Son W., Lee S. (2020). A deep motion sickness predictor induced by visual stimuli in virtual reality. IEEE Trans. Neural Netw. Learn. Syst..

[130] Tian N., Lopes P., Boulic R. (2022). A review of cybersickness in head-mounted displays: Raising attention to individual susceptibility. Virtual Real..

[131] McMahan R.P., Bowman D.A., Zielinski D.J., Brady R.B. (2012). Evaluating display fidelity and interaction fidelity in a virtual reality game. IEEE Trans. Vis. Comput. Graph..

[132] Ragan E.D., Bowman D.A., Kopper R., Stinson C., Scerbo S., McMahan R.P. (2015). Effects of field of view and visual complexity on virtual reality training effectiveness for a visual scanning task. IEEE Trans. Vis. Comput. Graph..

[133] Wen E., Gupta C., Sasikumar P., Billinghurst M., Wilmott J., Skow E., Dey A., Nanayakkara S. (2024). VR.net: A real-world dataset for virtual reality motion sickness research. IEEE Trans. Vis. Comput. Graph..

[134] Jennett C., Cox A.L., Cairns P., Dhoparee S., Epps A., Tijs T., Walton A. (2008). Measuring and defining the experience of immersion in games. Int. J. Hum.-Comput. Stud..

[135] Lapitan D.G., Rogatkin D.A., Molchanova E.A., Tarasov A.P. (2024). Estimation of phase distortions of the photoplethysmographic signal in digital IIR filtering. Sci. Rep..

[136] Privratsky A.A., Bush K.A., Bach D.R., Hahn E.M., Cisler J.M. (2020). Filtering and model-based analysis independently improve skin-conductance response measures in the fMRI environment: Validation in a sample of women with PTSD. Int. J. Psychophysiol..

[137] Richman J.S., Moorman J.R. (2000). Physiological time-series analysis using approximate entropy and sample entropy. Am. J. Physiol.-Heart Circ. Physiol..

[138] Posada-Quintero H.F., Florian J.P., Orjuela-Cañón Á.D., Chon K.H. (2016). Highly sensitive index of sympathetic activity based on time-frequency spectral analysis of electrodermal activity. Am. J. Physiol.-Regul. Integr. Comp. Physiol..

[139] Malik M., Bigger J.T., Camm A.J., Kleiger R.E., Malliani A., Moss A.J., Schwartz P.J. (1996). Heart rate variability: Standards of measurement, physiological interpretation, and clinical use. Eur. Heart J..

[140] Ciccone A.B., Siedlik J.A., Wecht J.M., Deckert J.A., Nguyen N.D., Weir J.P. (2017). Reminder: RMSSD and SD1 are identical heart rate variability metrics. Muscle Nerve.

[141] Brennan M., Palaniswami M., Kamen P. (2002). Do existing measures of Poincare plot geometry reflect nonlinear features of heart rate variability?. IEEE Trans. Biomed. Eng..

[142] Toichi M., Kubota Y., Murai T., Kamio Y., Sakihama M., Toriuchi T., Inakuma T., Sengoku A., Miyoshi K. (1999). The influence of psychotic states on the autonomic nervous system in schizophrenia. Int. J. Psychophysiol..

[143] Ishchenko A., Shev’ev P. (1989). Automated complex for multiparameter analysis of the galvanic skin response signal. Biomed. Eng..

[144] Greco A., Valenza G., Scilingo E.P. (2016). Modeling for the Analysis of the EDA. Advances in Electrodermal Activity Processing with Applications for Mental Health.

[145] Salvucci D.D., Goldberg J.H. Identifying fixations and saccades in eye-tracking protocols. Proceedings of the Symposium on Eye Tracking Research & Applications (ETRA).

[146] Tobii Tobii Pro Lab Gaze Filter. https://connect.tobii.com/s/article/Gaze-Filter-functions-and-effects?language=en_US.

[147] Golding J.F. (2006). Predicting individual differences in motion sickness susceptibility by questionnaire. Personal. Individ. Differ..

[148] Funder D.C., Ozer D.J. (2019). Evaluating effect size in psychological research: Sense and nonsense. Adv. Methods Pract. Psychol. Sci..

