# Supplementary material for: A Study on Autonomous Driving Motion Sickness from the Perspective of Multimodal Human Signals
Source: Sensors (Basel). 2026 Mar 6;26(5):1675. doi: 10.3390/s26051675 (PMC12987010; doi:10.3390/s26051675)
Supplement: Supplementary file 1 [file sensors-26-01675-s001.zip › sensors-4126083-supplementary.pdf]

sensors

Supplementary material

A study on autonomous driving motion sickness from the perspective of multimodal human signals

Su Young Kim[1] and Yoon Sang Kim[1,2,*]

[1] BioComputing Lab, Department of Computer Engineering, Korea University of Technology and Education (KOREATECH), Cheonan 31253, South Korea
[2] Institute for Bioengineering Application Technology, Korea University of Technology and Education (KOREATECH), Cheonan 31253, South Korea
* Correspondence: yoonsang@koreatech.ac.kr

Section S1. Detailed human-signal features by sensor domain in the HS-Set

Table S1. Specific search queries and results for each database.

Database	Search Query	Retrieved Records	Included Studies
IEEE Xplore	(("motion sickness" OR "cybersickness" OR "VIMS" OR "VR sickness") AND ("evaluation" OR "assessment" OR "quantification") AND ("physiological" OR "physiology" OR "biosignal" OR "biometric" OR "objective"))	113	25
Web of Science	(("motion sickness" OR "cybersickness" OR "VIMS" OR "VR sickness") AND ("evaluation" OR "assessment" OR "quantification") AND ("physiological" OR "physiology" OR "biosignal" OR "biometric" OR "objective"))	169	35
ACM Digital Library	[[All: "motion sickness"] OR [All: "cybersickness"] OR [All: " VIMS "] OR [All: " VR sickness"]] AND [[All: "evaluation"] OR [All: "assessment"] OR [All: "quantification"]] AND [[All: "physiological"] OR [All: "physiology"] OR [All: "biosignal"] OR [All: "biometric"] OR [All: "objective"]]	686	22

Section S1.1. Electrocardiogram and Photoplethysmogram

Although Electrocardiogram (ECG) and Photoplethysmogram (PPG) are based on different principles, the two signals are similar in that they both reflect cardiovascular activity and indirectly characterize cardiac dynamics. Some derived features, including heart rate (HR) and HR variability (HRV), which are the two most frequently used features in the HS-Set, can be calculated from both the ECG and PPG. Owing to this similarity, most studies in the HS-Set used either ECG or PPG rather than both; three studies used both ECG and PPG [36,57,60], 19 studies used only ECG, and 13 used only PPG.

The features used in this domain are listed in Table S2. The terms in Table S2 are defined as follows: *raw* features refer to the use of the windowed signal itself in machine learning (ML) / deep learning (DL) without extracting handcrafted features, *time-based statistics* features refer to statistical metrics such as mean, variance, kurtosis, skewness, maximum, and minimum, extracted from the raw or unspecified filtered signal in the time domain, and *simple frequency* features refer to the frequency-domain metrics transformed from the raw or unspecified filtered signal, such as the power spectral density (PSD).

Table S2. Summary of ECG and PPG features (not mutually exclusive).

Feature [1]	Description [2]	Count	Feature [1]	Description [2]	Count
HR	heart rate	19	Time-based statistics	time-domain statistical metrics from the raw/unspecified signal	3
HRV	heart rate variability, IBI-derived metrics (e.g., SDNN, RMSSD)	11	CVT	cardiac vagal tone, activity of the parasympathetic nervous system	2
IBI	inter-beat interval	7	CSI	cardiac sympathetic index, relative activity of the sympathetic nervous system	2
Raw	windowed signal itself	6	Poincaré-related	Poincaré plot metrics (e.g., SD 1, SD 2)	2
LF	spectral metrics in the low-frequency band	5	BR	breath rate	1
LF/HF	ratio of LF to HF	5	CSI/CVT	ratio of CSI to CVT	1
HF	spectral metrics in the high-frequency band	4	RPDE	recurrence period density entropy, signal periodicity and recurring patterns	1
Simple frequency	frequency-domain metrics transformed from the raw/unspecified signal	4			

[1] Conceptually similar derived metrics are grouped under a broader category.
[2] SD = Standard deviation, SDNN = SD of normal-to-normal intervals, RMSSD = Root mean square of successive differences.

Section S1.2. Electrodermal activity

Electrodermal activity (EDA), also known as galvanic skin response (GSR), has been widely used in several studies because the sympathetic nervous system is activated when motion sickness (MS) occurs [68]; it is the most frequently used domain when the ECG and PPG domains are considered separately. Its popularity in MS research can be attributed to the convenience of wearing the sensor at an accessible location, such as the wrist, compared to other sensors that are uncomfortable to wear. The features used in this domain are listed in Table S3, with *time-based statistics* being the most frequently used.

Table S3. Summary of EDA features (not mutually exclusive).

Feature [1]	Description [2]	Count	Feature [1]	Description [2]	Count
Time-based statistics	time-domain statistical metrics from the raw/unspecified signal	15	Simple frequency	frequency-domain metrics transformed from the raw/unspecified signal	2
Raw	windowed signal itself	9	3 intrinsic mode function	three signals extracted via EMD	1
SCL	skin conductance level, a slowly varying time-domain component (tonic)	5	HF	spectral metrics in the high-frequency band	1
SCR	skin conductance response, a rapidly varying time-domain component (phasic)	5	LF	spectral metrics in the low-frequency band	1

[1] Conceptually similar derived metrics are grouped under a broader category.
[2] EMD = Empirical Mode Decomposition.

Section S1.3. Eye

Eye data encompass both physiological (e.g., pupil diameter) and behavioral (e.g., gaze direction) features. Traditionally, these data were collected by processing images captured using externally mounted cameras or wearable camera sensors. Following the

introduction and widespread availability of the HTC VIVE Pro Eye, an eye-tracking HMD, many modern HMDs currently support eye tracking as a native feature. Since these HMDs allow for the extraction of eye features without additional equipment, they are mainly used in visually induced MS (VIMS) research involving VR HMDs. As listed in Table S4, the openness/blink, gaze movement, and pupil diameter features were frequently used. This is likely due to the software development kits (SDKs) of devices such as the HTC VIVE Pro Eye, which support these measurements.

Table S4. Summary of Eye features (not mutually exclusive).

Feature [1]	Description	Count	Feature [1]	Description	Count
Gaze movement	gaze motion metrics (e.g., direction, velocity)	13	GDC	gaze distance-to-center, an indicator of how far the gaze is from a defined center	2
Openness/ blink	eyelid openness and blink state	11	Slow phase velocity	gaze velocity during the slow phase of nystagmus	2
Pupil diameter	–	10	Heatmap entropy	entropy derived from a gaze heatmap	1
Pupil movement	pupil motion metrics (e.g., position, speed)	6	Simple frequency	frequency-domain metrics transformed from the raw/ unspecified signal	1
Saccade	rapid gaze movements	4	Eye-head coordination	degree of alignment between gaze and head movements	1
Fixation	slow gaze movements	4	Optokinetic nystagmus	reflexive eye oscillations induced by visual motion	1
Conver-gence distance	distance to the fixation point where eyes converge	4	Rhythm coherence	consistency in the rhythm of changes in the pupil-size	1
Path	gaze trajectory (length, etc.)	2	Vestibular parameter	vestibular variables derived from eye data	1
VOR	vestibulo-ocular reflex, eye movements compensating head motion, gain = eye/head velocity	2			

[1] Conceptually similar derived metrics are grouped under a broader category.

Section S1.4. Electroencephalogram

Although electroencephalogram (EEG) sensors are inconvenient to wear, they have been continuously used as an analytical tool across various fields because of their advantage of measuring neural activity. Most studies in the HS-Set analyzed EEG by decomposing the measured brainwaves into five frequency bands (α, β, γ, θ, and δ), as listed in Table S5. Because the frequency ranges of these five bands are nearly identical across studies, this decomposition method has become the de facto standard for EEG analysis. The raw features used in ML or DL are also frequently employed, whereas other features are less commonly used.

Table S5. Summary of EEG features (not mutually exclusive).

Feature [1]	Description	Count	Feature [1]	Description	Count
Standard frequency	frequency-domain metrics decomposed into the α, β, γ, θ, and δ bands	15	Power ratio	derived metrics based on the power ratio between bands.	1
Raw	windowed signal itself.	7	Excitement	indicator of a positive arousal state	1

Simple frequency	frequency-domain metrics transformed from the raw/ unspecified signal	3	Expanded frequency	frequency-domain metrics expanded from the standard frequency bands	1
FBN	functional brain network, functional connectivity across brain regions	2			

[1] Conceptually similar derived metrics are grouped under a broader category.

Section S1.5. Head

This domain includes data pertaining to the head that are tracked using externally mounted cameras, inertial measurement units (IMUs), or HMDs. As listed in Table S6, most studies used movement features. Except for the study by Sugiura *et al*. [95], these studies were identified as VIMS studies using VR HMDs. This finding suggests that head movement can be a key contributor to VIMS.

Table S6. Summary of Head features (not mutually exclusive).

Feature [1]	Description	Count	Feature [1]	Description	Count
Movement	head motion metrics (e.g., position, rotation, velocity, angular velocity)	16	Gravito-inertial force angle	angle between the gravito-inertial force and the head-vertical axis	1
Geographic coordinate	head orientation transformed into spherical (latitude–longitude) coordinates	2	Simple frequency	frequency-domain metrics transformed from the raw/ unspecified signal	1
Facial color	–	1			

[1] Conceptually similar derived metrics are grouped under a broader category.

Section S1.6. Respiration

When humans breathe, respiratory sinus arrhythmia causes HR to increase during inhalation and decrease during exhalation. Some respiration (RSP) features, such as the breath rate (BR), can be calculated using ECG/PPG data. However, some studies collected RSP data using dedicated sensors, such as pressure sensors attached to the abdomen or chest, instead of relying on PPG. The most commonly used derived feature was BR, which appeared in six studies [27,33,42,57,58,60]. Time-based statistical features, such as respiratory amplitude, were also frequently used in four studies [33,47,57,60]. Other features were rare, with only one study using raw features [36] and simple frequency features using a short-time fast Fourier transform [29].

Section S1.7. Assessment test

Some studies indirectly utilized physiological and behavioral indicators through assessment tests. Since each test was chosen based on the research purpose, no frequently used tests were identified. The head impulse test (HIT) was used in three studies to assess VOR, including functional and video versions [26,31,47]. The sensory organization test (SOT) was used in three studies to assess balance, including the head-shake SOT [30,47,98]. The videonystagmography test was used in two studies to assess oculomotor and vestibular functions by measuring nystagmus [30,65]. Six other tests were used once each: Andre and Coutellier [44] utilized Romberg's posture test for balance indicators, Kim *et al.* [47] utilized the near point of accommodation and convergence tests for visual function assessment indicators, and Berton et al. [80] utilized the critical flicker-fusion frequency test for visual fatigue indicators. Aydin et al. [30] used the near visual acuity test for vision indicators and the visual evoked potential test for visual function indicators.

Section S1.8. Skin temperature

The activation of the sympathetic nervous system due to MS also affect the skin temperature (SKT) [47,68]. However, because of the slow- and low-frequency characteristics

of SKT, this domain lacks real-time responsiveness and has been used in relatively few studies. The most commonly used SKT features were time-based statistics, which appeared in five studies [33,37,47,57,65]. Other features were rare, with only two studies using raw features [61,62] and one using simple frequency features [62].

Section S1.9. Electrogastrogram

Since nausea and vomiting are major symptoms of MS, some studies used electrogastrogram (EGG) to directly investigate these symptoms, despite the inconvenience of electrode placement. EGG has major frequency band ranges including tachygastria, bradygastria, and normogastria. However, as listed in Table S7, only a single study was identified for each band except for tachygastric. Additionally, unlike EEG and EDA, frequency band decomposition features were not primarily used, and time-based statistics and simple frequency features were mainly employed.

Table S7. Summary of EGG features (not mutually exclusive).

Feature [1]	Description [2]	Count	Feature [1]	Description [2]	Count
Time-based statistics	time-domain statistical metrics from the raw/unspecified signal	4	Normogastria	band component, corresponding to the normal gastric slow-wave rhythm	1
Simple frequency	frequency-domain metrics transformed from the raw/ unspecified signal	4	Frequency spectrum density	peak-referenced distribution of spectral power across frequencies	1
Tachygastria	high-frequency band component (related stress and nausea)	3	Bradygastria	low-frequency band component (related relaxation and rest)	1
Crest factor	ratio of peak to root mean square	2	Poincaré-related	Poincaré plot metrics (e.g., SD 1, SD 2)	1
Dominant frequency	dominant frequency component	2	Power ratio	derived metrics based on the power ratio between bands	1
Arrhythmia	arrhythmic gastric activity	1			

[1] Conceptually similar derived metrics are grouped under a broader category.

[2] SD = Standard deviation.

Section S1.10. Center of pressure

The center of pressure (CoP) refers to the balance data derived from pressure changes when a person stands or moves. The postural instability theory, one of the MS hypotheses, posits that MS is induced when the ability to maintain balance using the ankle, knee, and hip muscles in response to movement becomes unstable (Riccio and Stoffregen, 1991 [a]). Based on this theory, some studies used this domain to investigate MS, focusing on two main feature types: movement features (e.g., displacement, angle, velocity, and trajectory) and area-derived features. Five studies used movement features [35,65,98,100,101], and five studies used area-derived features [35,65,98,100,102].

[a] Riccio, G.E.; Stoffregen, T.A. An ecological theory of motion sickness and postural instability. *Ecol. Psychol.* **1991**, *3*, 195–240. https://doi.org/10.1207/s15326969eco0303_2.

Section S1.11. Electromyogram

The sensory conflict theory, a leading mechanistic hypothesis of MS, posits that MS is induced by mismatches between visual, vestibular, and somatosensory cues (Reason and Brand, 1975 [b]; Oman, 1990 [c]; Bles *et al.*, 1998 [d], Bos *et al.*, 2008 [e]). Many studies in the HS-Set focused on the visual and vestibular senses in MS, but some studies used electromyogram (EMG) to extend the investigation to the somatosensory system. EMG was also used for analyzing the physical fatigue accompanying MS. Although one study defined features in detail according to the placement of the EMG sensors [55], we broadly

categorized the features used into time-based statistics and simple frequency. Five studies used time-based statistical features [35,47,55,57,80], and one used simple frequency features [55].

[b] Reason, J.T.; Brand, J.J. *Motion sickness*; Academic press: Oxford, England, 1975.

[c] Oman, C.M. Motion sickness: A synthesis and evaluation of the sensory conflict theory. *Can. J. Physiol. Pharmacol.* **1990**, *68*, 294–303. https://doi.org/10.1139/y90-044.

[d] Bles, W.; Bos, J.E.; De Graaf, B.; Groen, E.; Wertheim, A.H. Motion sickness: Only one provocative conflict? *Brain Res. Bull.* **1998**, *47*, 481–487. doi:10.1016/S0361-9230(98)00115-4.

[e] Bos, J.E.; Bles, W.; Groen, E.L. A theory on visually induced motion sickness. *Displays* **2008**, *29*, 47–57. doi:10.1016/j.displa.2007.09.002.

Section S1.12. Miscellaneous sensor domains

The sensor domains with three or fewer cumulative studies in the HS-Set were functional near-infrared spectroscopy (fNIRS) data, functional magnetic resonance imaging (fMRI) data, blood pressure (BP), impedance cardiography (ICG) data, gait, body, and endocrine systems. Owing to the limited number of studies, they are summarized in Table S8 by the features used rather than by their frequencies.

Table S8. Summary of miscellaneous features (not mutually exclusive).

Domain	Features [1]	Description	Domain	Features [1]	Description
fNIRS	HbO	oxygenated hemoglobin	ICG	Cardiac output	amount of blood pumped per min from the heart
fNIRS	HbR	deoxygenated hemoglobin	ICG	Stroke volume	amount of blood pumped from the heart with each beat
fNIRS	HbT	total hemoglobin concentration change	Gait	Movement	gait motion metrics (e.g., position, angle, velocity)
fNIRS	Power spectral entropy	entropy of the power spectrum from brain-hemoglobin signals	Gait	Stance or swing	indicates whether the foot is on the ground
fMRI	BOLD	blood-oxygen-level-dependent, brain activation metrics (e.g., voxel/cluster contrasts)	Gait	Stride	–
fMRI	Brain connectivity	network of BOLD signals between brain regions	Body	Movement	body motion metrics
fMRI	Morphology	3D shape of the brain	Endocrine	Ghrelin	appetite-stimulating hormone
fMRI	Volumetry	volume of the brain	Endocrine	Vasopressin	antidiuretic hormone
BP	Time-based statistics	time-domain statistical metrics from the raw/unspecified signal			

[1] Conceptually similar derived metrics are grouped under a broader category.

Section S2. Technical details of the autonomous driving simulator

Section 2.1. Motion cueing algorithm

The classic washout filter method used in this study generates the position signal of the platform by applying second-order high-pass filters (HPF_2s) to the acceleration, a, and generates the rotational signal of the platform by merging the result of applying second-order low-pass filters (LPF_2s) to a with the result of applying first-order high-pass filters (HPF_1s) to the angular velocity, ω.

During the transformation process, the damping coefficient, ζ, for HPF_2 and LPF_2 was set to 1, consistent with the value used by Nahon and Reid (1990) [f], whereas the cutoff

frequencies were adjusted to match the motion platform used. Table S9 presents the cutoff frequencies and scale factors used for each filter.

Table S9. Cutoff frequencies and scale factors used in the MCA.

Filter [1,2]	Cutoff Frequency (HZ)	Scale Factor
HPF_1–yaw	0.2	2.5
HPF_1 –pitch	0.5	20
HPF_2–sway, surge	0.6	0.5
LPF_2	0.4	0.4
Tilt – pitch	-	2.5 rad
Tilt – roll	-	2.5 rad

[1] $HPF = s / (s+w)$, $HPF_2 = s^2 / (s^2 + 2w\zeta s + w^2)$, $LPF_2 = w^2 / (s^2 + 2w\zeta s + w^2)$, $w = 2\pi f_{cutoff}$, s = complex variable. [2] Road surface protrusions (e.g., speed bumps and potholes) and cross slopes were excluded, and thus the heave and roll filters were not used.

[f] Nahon, M.A.; Reid, L.D. Simulator motion-drive algorithms-a designer's perspective. *J. Guid. Control Dyn.* **1990**, *13*, 356–362. https://doi.org/10.2514/3.20557.

Section S2.2. Virtual environment and motion profile generation

The predefined motion profiles for each driving scenario, which consist of acceleration and angular velocity, were modeled using sinusoids. These models approximated the simulation results from the open-source virtual AV simulator CARLA (Dosovitskiy *et al.*, 2017 [g]). Because Unity outputs acceleration and angular velocity signals in discrete time, the MCA was implemented as discrete-time difference equations.

The trajectory of the virtual AV was generated through time integration, and the road and surrounding environment were formed based on this trajectory. Figure S1 shows the motion profiles and generated trajectories for each scenario (the straight constant velocity scenario was excluded because it generated no acceleration or angular velocity). Figure S2 shows the process of generating the road and surrounding environment based on vehicle trajectories.

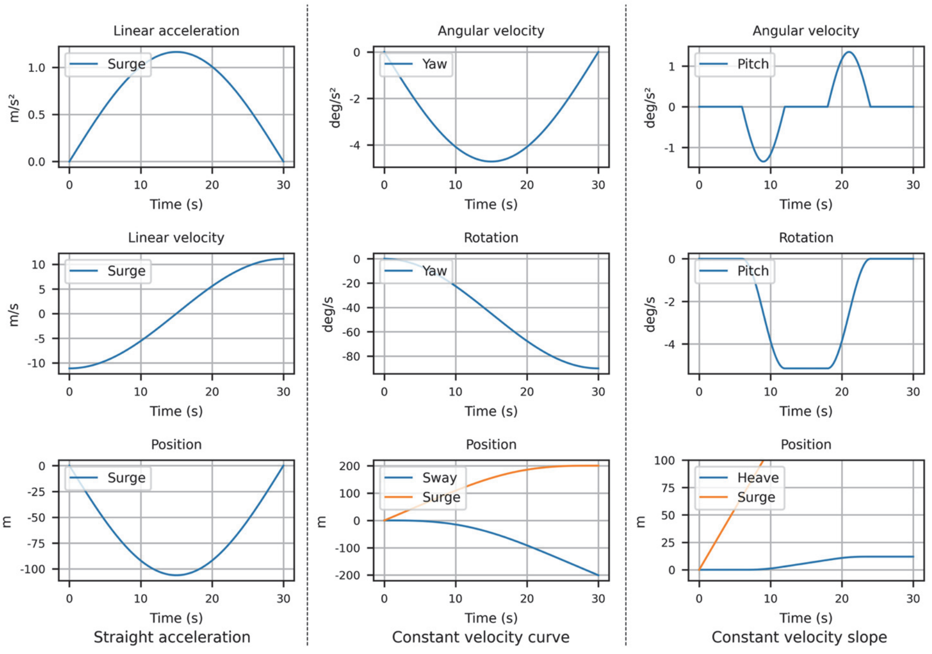

Figure S1. Motion profiles and vehicle trajectories for different driving scenarios.

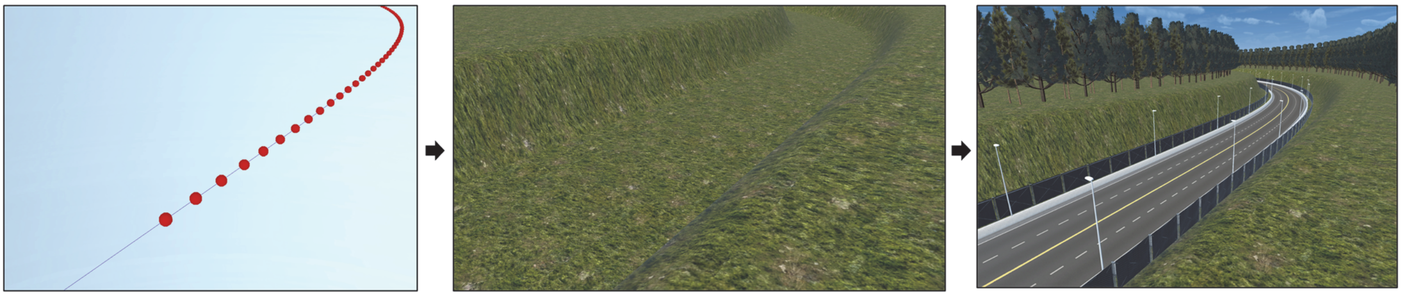

Figure S2. Process of road and surrounding environment generation based on vehicle trajectories.

[g] Dosovitskiy, A.; Ros, G.; Codevilla, F.; Lopez, A.; Koltun, V. CARLA: An open urban driving simulator. In Proceedings of the Conference on robot learning (CoRL), Mountain view, CA, USA, Nov. 13–15, 2017; pp. 1–16.

Section S3. Data synchronization and system I/O optimization

Continuous reception from multiple sensors could cause computational overload on the server, and the OS time resolution limit (15.6 ms ≈ 64 Hz) made it difficult to receive high-frequency signals directly. Therefore, the data from each sensor were bundled into chunks and transmitted at a rate of approximately 15–20 Hz. When the recording mode was activated, the receivers of the server unpacked the signals from these chunks and loaded them into sensor-specific queues in the data manager. Every second, data were extracted from all queues, time-aligned to a common clock referenced to the EEG stream (500 Hz, the highest rate), and stored. To prevent file I/O bottlenecks during this process, the data were first placed in a separate queue and written to a file after more than 20 s of data accumulation. Figure S3 shows the operator console, which allowed real-time monitoring of multimodal signals and remote control of the recording.

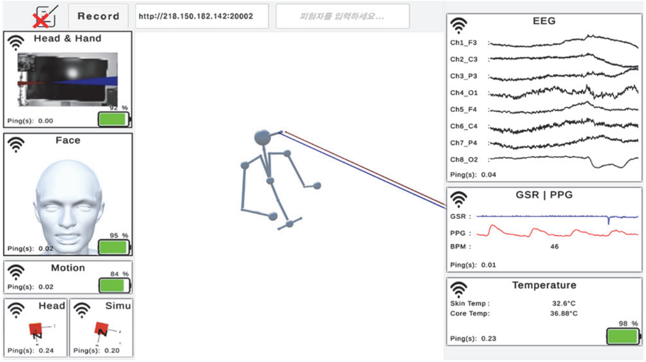

Figure S3. Operator console interface for real-time monitoring and remote recording.

Section S3. EEG preprocessing filter settings in the HS-Set

Table S10. Lower and upper cutoff frequencies for EEG preprocessing band-pass filters in the HS-Set.

EEG study in the HS-Set	Lower cutoff [1]	Upper cutoff [2]	EEG study in the HS-Set	Lower cutoff [1]	Upper cutoff [2]
Hu *et al.* [89]	1	40	Liu *et al.* [84]	0.5	50
Hua *et al.* [83]	0.5	45	Liu *et al.* [87]	0.1	50
Jang *et al.* [92]	0.1	100	Oh and Son [40]	1	59
Jeong *et al.* [93]	4	40	Recenti *et al.* [55]	0.4	40
Lee *et al.* [38]	0.5	50	Sameri *et al.* [39]	1	-

Li *et al.* [85]	1	40	Woo *et al.* [88]	0.5	100
Lim *et al.* [90]	0.1	100	Xu *et al.* [91]	1	50

[1] The most frequent value is 1 Hz (n = 5), followed by 0.5 Hz (n = 4).

[2] The most frequent values are 40 Hz (n = 4) and 50 Hz (n = 4), followed by 100 Hz (n = 3). One study did not report an upper cutoff.

Table S11. Lower and upper cutoff frequencies for the EEG decomposition bands (δ-γ) in the HS-Set.

No.	δ band (cutoff)		θ band (cutoff)		α band (cutoff)		β band (cutoff)		γ band (cutoff)	
	Lower	Upper	Lower	Upper	Lower	Upper	Lower	Upper	Lower	Upper
Hu *et al.* [89]	0.5	4	4	8	8	13	13	30	–	–
Hua *et al.* [83]	0.5	4	4	8	8	12	15	30	30	45
Jang *et al.* [92]	1	3.5	4	7.5	8	12	–	–	–	–
Jeong *et al.* [93]	–	–	4	8	8	12	12	25	25	45
Kim *et al.* [54]	1	3	4	7	8	13	14	30	31	50
Lee *et al.* [38]	0.2	4	4	8	8	13	13	30	30	50
Li *et al.* [85]	1	4	4	8	8	13	13	28	28	40
Lim *et al.* [90]	–	4	4	8	8	12	12	30	30	–
Liu *et al.* [84]	1	4	4	8	8	12	–	–	–	–
Liu *et al.* [87]	0.	4	4	8	8	13	13	30	30	45
Su and Jia [37]	1	3	4	7	8	13	14	30	31	50
Sameri *et al.* [39]	–	–	4	7	8	13	14	29	30	47
Oh and Son [40]	1	3.99	4	7	8	15	16	31	32	59
Recenti *et al.* [55]	0.5	4	4	8	8	13	13	35	35	40
Woo *et al.* [88]	1	4	–	–	–	–	–	–	–	–
Mode (count)	1 (7)	4 (9)	4 (14)	8 (9)	8 (14)	13 (8)	13 (5)	30 (7)	30 (5)	45, 50 (3)
